# TOR complex 2 (TORC2) signaling and the ESCRT machinery cooperate in the protection of plasma membrane integrity in yeast

**DOI:** 10.1074/jbc.RA120.013222

**Published:** 2020-07-01

**Authors:** Oliver Schmidt, Yannick Weyer, Simon Sprenger, Michael A. Widerin, Sebastian Eising, Verena Baumann, Mihaela Angelova, Robbie Loewith, Christopher J. Stefan, Michael W. Hess, Florian Fröhlich, David Teis

**Affiliations:** 1 Institute for Cell Biology, Biocenter, Medical University of Innsbruck, Innsbruck, Austria; 2 Department of Biology/Chemistry, University of Osnabrück, Osnabrück, Germany; 3 Cancer Evolution and Genome Instability Laboratory, Francis Crick Institute, London, United Kingdom; 4 Department of Molecular Biology, University of Geneva, Geneva, Switzerland; 5 MRC Laboratory for Molecular Cell Biology, University College London, London, United Kingdom; 6 Institute for Histology and Embryology, Medical University of Innsbruck, Innsbruck, Austria

**Keywords:** calcineurin, membrane stress, TORC2, ORMDL family, endosome and Golgi-associated degradation (EGAD), sphingolipid, membrane, stress, endosomal sorting complexes required for transport (ESCRT), mTOR complex (mTORC), ORMDL family

## Abstract

The endosomal sorting complexes required for transport (ESCRT) mediate evolutionarily conserved membrane remodeling processes. Here, we used budding yeast (*Saccharomyces cerevisiae*) to explore how the ESCRT machinery contributes to plasma membrane (PM) homeostasis. We found that in response to reduced membrane tension and inhibition of TOR complex 2 (TORC2), ESCRT-III/Vps4 assemblies form at the PM and help maintain membrane integrity. In turn, the growth of ESCRT mutants strongly depended on TORC2-mediated homeostatic regulation of sphingolipid (SL) metabolism. This was caused by calcineurin-dependent dephosphorylation of Orm2, a repressor of SL biosynthesis. Calcineurin activity impaired Orm2 export from the endoplasmic reticulum (ER) and thereby hampered its subsequent endosome and Golgi-associated degradation (EGAD). The ensuing accumulation of Orm2 at the ER in ESCRT mutants necessitated TORC2 signaling through its downstream kinase Ypk1, which repressed Orm2 and prevented a detrimental imbalance of SL metabolism. Our findings reveal compensatory cross-talk between the ESCRT machinery, calcineurin/TORC2 signaling, and the EGAD pathway important for the regulation of SL biosynthesis and the maintenance of PM homeostasis.

Eukaryotic cells harmonize lipid homeostasis and protein homeostasis (proteostasis) to maintain the integrity of their membranes. This requires the balanced synthesis of proteins and lipids and their selective degradation. How these processes are coordinated is only partially understood.

Selective membrane protein degradation is mediated through different ubiquitin-dependent degradation pathways. In the endoplasmatic reticulum (ER), the ER-associated degradation (ERAD) machinery ubiquitinates membrane proteins for retro-translocation into the cytoplasm and subsequent proteasomal degradation (1). Proteins in post-ER organelles are degraded by different pathways. The degradation of most membrane proteins of the PM, Golgi, or endo- and lysosomal membranes occurs inside lysosomes (vacuoles in yeast) and depends on the ESCRT machinery (2, 3). The ESCRT machinery sequesters ubiquitinated cargo proteins on the endosome surface and catalyzes the budding of small cargo-laden vesicles into the lumen of the endosome (4). Membrane remodeling is accomplished by the transient assembly of ESCRT-III complexes together with the AAA-ATPase Vps4. This process generates multivesicular bodies (MVB), which subsequently fuse with the lysosome/vacuole. Loss of function of the ESCRT machinery leads to the formation of aberrant, peri-vacuolar endosomal structures called class E compartments, where membrane protein cargoes of the MVB pathway accumulate. The ESCRT machinery is additionally involved in many other cellular membrane remodeling events (for a recent review on ESCRT functions, see Refs. 3 and 5), and also repairs ruptured lysosomes, maintains the integrity of the nuclear envelop and patches up holes in the plasma membrane (3).

Besides the ESCRT machinery, the endosome and Golgi-associated degradation (EGAD) pathway operates on a different set of membrane proteins that are exported from the ER. EGAD substrates are ubiquitinated on Golgi and endosomes and then extracted from membranes for proteasomal degradation (6). Ubiquitination is mediated by the defective in sterol-responsive element binding protein cleavage (Dsc) complex, a multisubunit transmembrane ubiquitin ligase reminiscent of the ERAD complexes, and extraction is executed by Cdc48 (6, 7). A regulated EGAD substrate is the transmembrane protein Orm2 (6). Orm2 is part of the multisubunit SPOTS complex in the ER, which consists of Orm1/2, the catalytic subunits of the serine-palmitoyl-CoA transferase (SPT, Lcb1, and Lcb2), and the regulatory factors Tsc3 and Sac1 (8). The SPT mediates sphingoid long chain base (LCB) synthesis and is rate-limiting for sphingolipid metabolism. The Orm1/2 proteins repress SPT activity, which is important to prevent the toxic accumulation of LCBs. The de-repression of SPT activity is mediated by phosphorylation of the Orm1/2 proteins in their N-terminal cytosolic domain by the AGC-family serine-threonine protein kinases Ypk1 and its paralogue Ypk2 (9). Phosphorylated Orm2 is exported from the ER and subsequently degraded by EGAD (6). Failure to phosphorylate and/or to degrade Orm2 via EGAD critically impairs SL homeostasis. The ORMDL protein family is evolutionary conserved and their accumulation is linked to human diseases (10–12).

Ypk1/2 are the orthologues of the mammalian serum- and glucocorticoid-induced protein kinase SGK1 (9). In yeast, Ypk1/2 are activated through phosphorylation by phosphoinositide-dependent kinase (Pkh1/2) and the target of rapamycin complex 2 (TORC2) (13). TORC2 is an essential, PM-localized protein kinase whose activity responds to membrane stress such as changes in PM tension (14, 15) and sphingolipid levels (16). How TORC2 senses membrane stress is largely unknown (14, 15, 17). The essential function of the TORC2-Ypk1 signaling axis is the phosphorylation of Orm1/2 for the controlled de-repression of LCB synthesis (9, 18). Ypk1 also stimulates ceramide synthesis (19), which helps to control flux of LCBs into ceramides and complex SL. Thus, TORC2 signaling responds to tensile stress at the PM and adjusts via Ypk1/2 sphingolipid synthesis and other processes to maintain PM homeostasis (14, 15, 18–24).

Regulatory roles of ERAD and EGAD in maintaining lipid metabolism and membrane proteostasis have been described. How the ESCRT machinery contributes to the regulation of membrane homeostasis is unclear and inter-relationships between these membrane proteostasis networks have not been elucidated. Here we show that ESCRT-III and Vps4 assemble at the PM in response to reduced membrane tension or TORC2 inhibition and help to preserve membrane integrity. In turn, ESCRT mutants are hyper-sensitive to PM stress conditions and critically rely upon TORC2-Ypk1 signaling to counteract membrane stress that is caused by accumulation of Orm2 at the ER. In ESCRT mutants, calcineurin phosphatase activity hampers Orm2 ER export and subsequent EGAD-dependent degradation. Inhibiting dephosphorylation of Orm2 and promoting its degradation by EGAD relieves the dependence of ESCRT mutants on TORC2-Ypk1 signaling. This indicates that the accumulation of Orm2 at the ER is a major membrane stressor. Our results reveal important links between the ESCRT machinery, TORC2-Ypk1–dependent membrane stress signaling and the EGAD pathway, which cooperatively protect PM integrity.

## Results

### ESCRT mutants depend on TORC2-Ypk1 signaling for cell growth and survival

The evolutionary conserved ESCRT complexes assemble into membrane remodeling machineries that catalyze the biogenesis and the repair of diverse organelles (3). Despite these key roles in generating and maintaining cellular membrane systems, ESCRT-deficient cells are viable. This implies that cells can at least in part compensate for the loss of ESCRT function. Understanding how cells tolerate loss of the ESCRT machinery would provide insight into adaptive mechanisms that mitigate cellular membrane stress.

**Figure 1.**
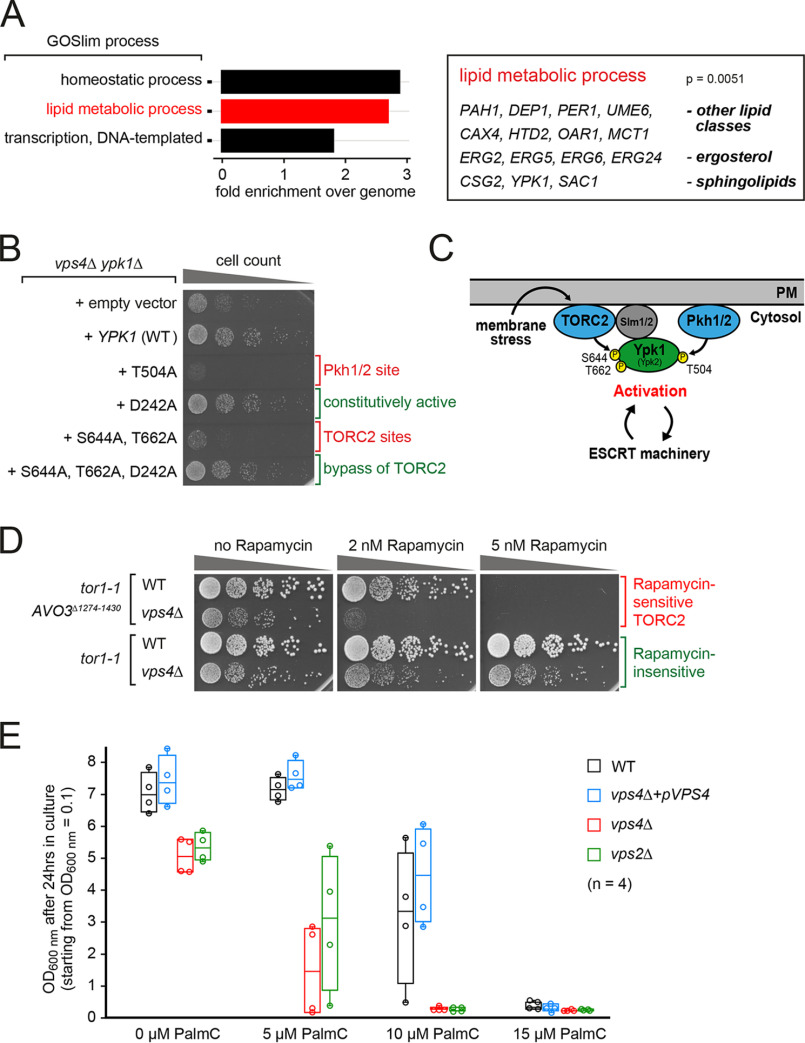
**ESCRT mutants depend on lipid metabolism and TORC2-Ypk1 signaling for growth and survival.** *A,* GO analysis for cellular processes of 119 genes that showed negative genetic interactions with *vps4*Δ (6). Only significantly enriched GO terms (*p* < 0.05) are shown. 15 genes comprised in the GO term lipid metabolic process are shown. Full GO analysis is described in Table S1. *B,* equal amounts of *vps4*Δ *ypk1*Δ double mutants expressing the indicated *YPK1* mutant plasmids (or empty vector) in serial dilutions were incubated on auxotrophic selection medium agar plates at 26 °C. *C,* schematic representation of the activation of the TORC2-Ypk1 signaling pathway in response to membrane stress and its putative interdependence with the ESCRT machinery. *Pkh1/2*, yeast orthologues of 3-phosphoinositide-dependent kinase 1. *D,* equal amounts of the indicated strains in serial dilutions were incubated on auxotrophic selection medium agar plates containing the indicated rapamycin concentrations at 26 °C. All WT strains are the respective *vps4*Δ strain re-expressing *VPS4* from plasmid. *E,* growth of the indicated strains in the presence of PalmC. Cells were inoculated to OD_600 nm_ = 0.1 in auxotrophic selection medium containing the indicated PalmC concentrations and grown for 24 h in 4 independent experiments. The *circles* indicate the individual measurements. See also Fig. S1 and Table S1.

To identify the processes that enable the survival of ESCRT-deficient cells, we used the budding yeast, *Saccharomyces cerevisiae*, as a model system and conducted a genome-wide synthetic genetic interaction screen (6). Gene ontology (GO) term enrichment analysis revealed “lipid metabolism” among the top ranked hits that are required for the growth and survival of ESCRT mutants (*vps4*Δ) (Fig. 1*A*, Table S1). This GO term included genes involved in the metabolism of various lipid classes, but predominantly enzymes mediating nonessential steps of sterol biosynthesis and genes regulating SL homeostasis. Sphingolipids and ergosterol are known to promote membrane rigidity and also to stabilize many membrane proteins (25, 26). In concert, these lipids might therefore become essential in ESCRT mutants to counteract membrane stress. We confirmed the synthetic growth defect of *vps4*Δ cells with four of these genes (*ypk1*Δ, *sac1*Δ, *csg2*Δ, *erg2*Δ) in a different genetic background (the SEY6210 strain) (Fig. S1*A*). These results suggested that the loss of ESCRT function rendered cells particularly sensitive to perturbations in membrane homeostasis.

We focused on the role of the protein kinase Ypk1 in ESCRT-deficient cells, because it functions directly downstream of TORC2 to maintain plasma membrane homeostasis (9, 14, 23, 27, 28). *YPK1* deletion (but not deletion of *YPK2*) also caused synthetic growth defects with ESCRT-II (*vps25*Δ) and ESCRT-III mutants (*vps2*Δ), but not with mutants of Chm7 (*chm7*Δ) (Fig. S1*B*), which recruits the ESCRT machinery to the ER and the inner nuclear membrane (29–31). This result suggested that Ypk1 was required to compensate for the loss of ESCRT functions that are independent of Chm7.

The growth of *vps4*Δ *ypk1*Δ double mutants was restored by re-expression of *VPS4* and *YPK1* from plasmids (Fig. S1*C*), but not by an additional copy of *YPK2* (32). Expression of a Ypk1 mutant (Ypk1 S644A,T662A) that can no longer be activated by TORC2 failed to rescue the growth defects of *vps4*Δ *ypk1*Δ (Fig. 1, *B* and *C*) or *vps2*Δ *ypk1*Δ (Fig. S1*D*) double mutants, similar to the catalytically dead T504A mutant. The constitutively active Ypk1 D242A mutant (9) restored normal growth of the double mutants, even when the TORC2-acceptor sites were mutated (Fig. 1*B*, Fig. S1*D*). Hence, TORC2-dependent Ypk1 activation was required in ESCRT mutants.

To further characterize the relationship between TORC2 signaling and the ESCRT machinery, we analyzed the growth of WT and ESCRT mutant cells, which were genetically engineered for the selective inhibition of TORC2 (*tor1-1 AVO3*^Δ1274-1430^) with rapamycin (33). At 2 nm rapamycin the growth of WT cells was only mildly affected (33), whereas the growth of *vps4*Δ mutants was already markedly reduced (Fig. 1*D*). TORC2 responds to perturbation of the PM, and an acute reduction of PM tension with palmitoyl-carnitine (PalmC) leads to transient TORC2 inactivation (15). Importantly, the growth of WT cells was not affected by low doses of PalmC (5 μm), suggesting that they were capable to maintain PM homeostasis, although the same PalmC concentration slowed down the growth of *vps4*Δ or *vps2*Δ cells (Fig. 1*E*). It seemed that ESCRT function contributed to viability upon reduction of PM tension and TORC2 signaling.

### ESCRT-III and Vps4 localize to plasma membrane structures upon TORC2 inhibition

**Figure 2.**
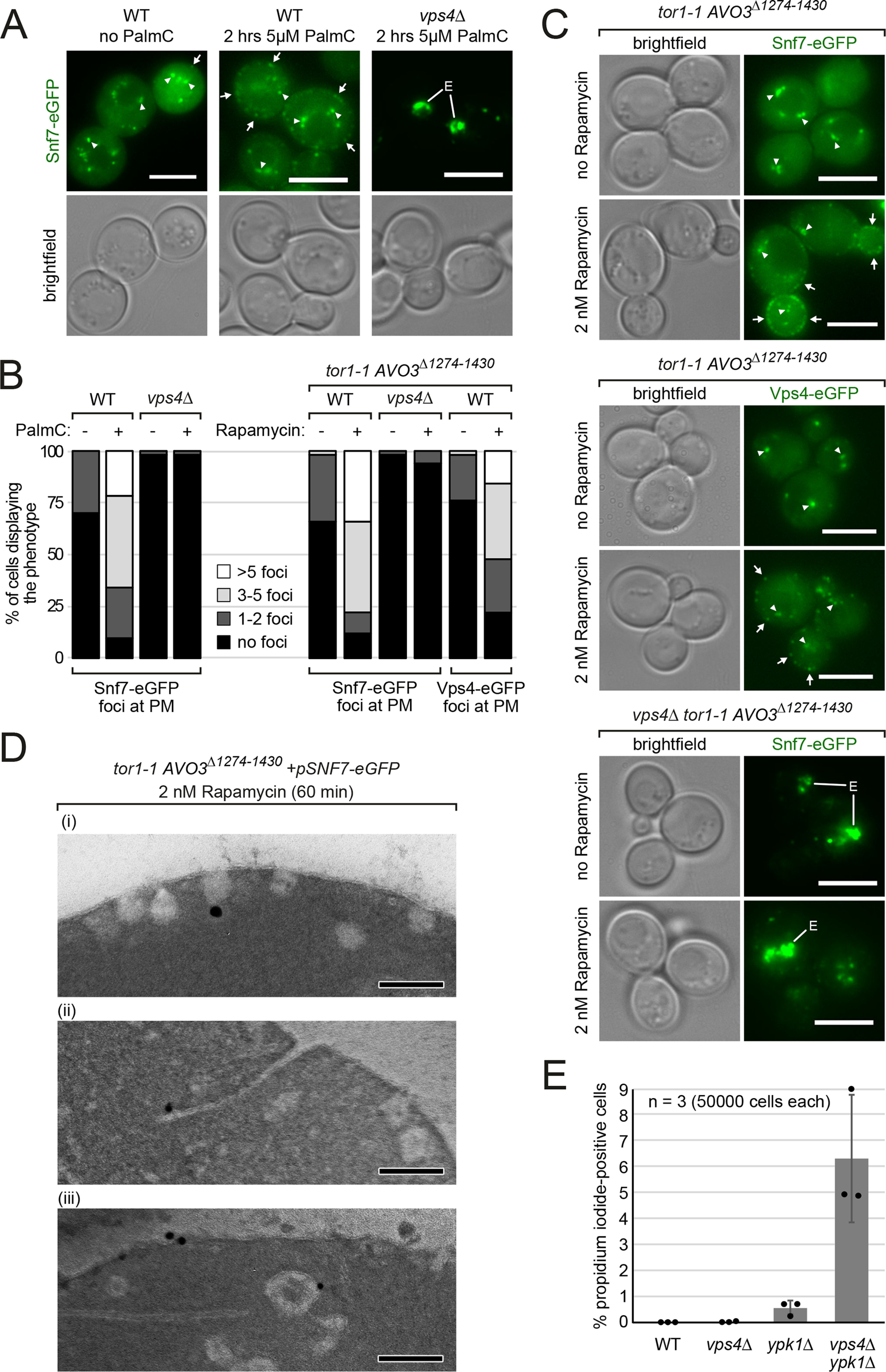
**ESCRT-III and Vps4 localize to plasma membrane structures upon TORC2 inhibition.** *A–D,* all WT strains are isogenic to the respective *vps4*Δ cells and re-express *VPS4* (or *VPS4-eGFP* where indicated) from a plasmid. *A,* epifluorescence and phase-contrast microscopy of WT (*vps4*Δ + *pVPS4*) and *vps4*Δ cells co-expressing Snf7- eGFP (*green*) from a centromeric plasmid and treated with 5 μm PalmC or vehicle (DMSO) for 2 h. *Arrowheads* indicate Snf7 on endosomes/MVBs and *arrows* indicate Snf7 assemblies at the PM. *Scale bars*, 5 μm. *B,* quantification of *A* and *C*. Subcortical eGFP dots in 50 cells of each genotype and condition were counted and grouped into the indicated cohorts. *C,* epifluorescence and phase-contrast microscopy of the indicated strains co-expressing *SNF7-eGFP* or expressing *VPS4-eGFP* from centromeric plasmids and treated with 2 nm rapamycin or vehicle (DMSO) for 2 h. *Arrowheads* indicate endosomes/MVBs and *arrows* indicate Snf7/Vps4 assemblies at the PM. *Scale bars*, 5 μm. *D,* high-pressure freezing, anti-GFP immunogold labeling, and transmission EM of the indicated cells treated with 2 nm rapamycin for 60 min. *Scale bars,* 200 nm. *E,* the indicated strains were grown into mid-log phase and stained with propidium iodide for 10 min. 50,000 cells were analyzed by flow cytometry. Data are presented as mean ± S.D. from 3 independent experiments.

We next asked how ESCRT-dependent processes might be correlated to tensile PM stress and TORC2 signaling. Therefore, we analyzed the localization of the major ESCRT-III subunit Snf7 in cells that were treated with PalmC at a concentration (5 μm) that did not affect the growth of WT cells (Fig. 1*E*). In untreated cells, Snf7-eGFP was mainly detected in the cytosol and on objects corresponding to endosomes and MVBs (4) (Fig. 2*A*, *arrowheads*). Upon addition of PalmC, we detected the formation of Snf7-eGFP foci at or close to the PM (Fig. 2*A*, *arrows*). After 2 h, at least three of these structures were observed in the majority of cells (66%) (Fig. 2, *A* and *B*).

Next, we tested if the localization of ESCRT-III to the PM was a consequence of TORC2 inactivation. We determined the localization of Snf7 and Vps4 upon sublethal inhibition of TORC2 signaling (2 nm rapamycin) (Fig. 1*D*). Snf7-eGFP and Vps4-eGFP assemblies formed at the PM in more than 75% of cells after 2 h of TORC2 inhibition (Fig. 2, *B* and *C*, *arrows*). One or two subcortical Snf7-eGFP and Vps4-eGFP dots but rarely more were also observed in untreated WT cells (in 22–34% of cells) (Fig. 2*B*). Thus, ESCRT-III assemblies formed at the PM in response to PalmC-induced membrane perturbations and upon TORC2 inhibition.

To morphologically analyze the identity of the Snf7-eGFP positive structures that emerge upon PM stress at the ultrastructural level, we performed cryofixation and immunogold EM (4). After 60 min of sublethal TORC2 inhibition, we observed numerous vesicular structures at or near the PM (Fig. 2*D*). PM-associated vesicles likely represented invaginations, possibly reflecting stalled endocytic events, because TORC2 signaling stimulates endocytosis on multiple levels (18, 20, 23). Snf7-eGFP localized to these invaginations (Fig. 2*D*) (i), to extended tubular invaginations (ii), as well as to flat areas and vesicular structures in close vicinity to the PM (iii).

The localization of Snf7-eGFP to PM foci in response to PalmC treatment or direct TORC2 inhibition required the activity of Vps4. In *vps4*Δ mutants, Snf7-eGFP remained associated with class E compartments (4) (Fig. 2, *A–C*). Of note, the growth of *vps4*Δ cells was already severely reduced under these conditions (Fig. 1, *D* and *E*). Hence the localization of ESCRT-III/Vps4 to the PM might help to mitigate PM stress and prevent cell injuries.

To assess PM integrity, we used propidium iodide (PI) staining. PI is membrane impermeable and only enters yeast cells when the sealing of the PM is corrupted. In WT cells, but also in *vps4*Δ and *ypk1*Δ single mutants, the frequency of PI-positive cells was low (Fig. 2*E*). Yet, the frequency of PI-positive cells strongly increased in *vps4*Δ *ypk1*Δ double mutants (Fig. 2*E*). Thus, the chronic inactivation of Ypk1 (chronic TORC2 inactivation is lethal) in *vps4*Δ mutants dramatically increased the frequency of PM integrity defects. These results demonstrated that loss of Ypk1 signaling combined with the inability to form ESCRT-III/Vps4 assemblies at the PM severely compromised membrane integrity and cell survival.

### TORC2-Ypk1 signaling is elevated in ESCRT mutants

**Figure 3.**
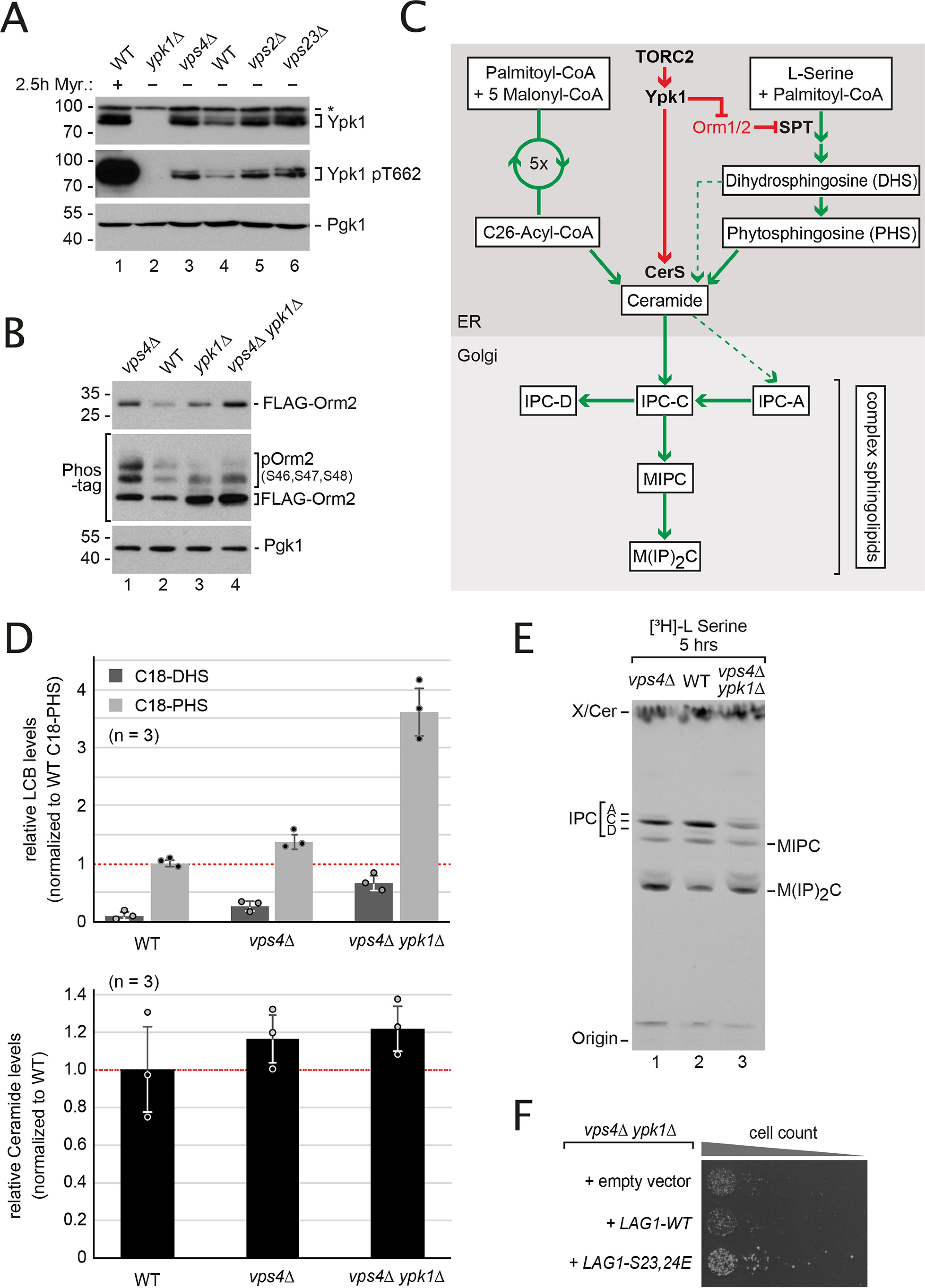
**Elevated TORC2-Ypk1 signaling prevents detrimental SL imbalance in ESCRT mutants.** *A,* SDS-PAGE and Western blotting analysis with the indicated antibodies of total yeast lysates from WT cells and the indicated mutants. *Myr*., myriocin (1.5 μm, 2.5 h). *B,* SDS-PAGE or Phos-tag SDS-PAGE and Western blotting analysis with the indicated antibodies of total yeast lysates from WT cells and the indicated mutants expressing *FLAG-ORM2* (in *orm2*Δ background). Quantification in Fig. S2*F*. *C,* schematic representation of SL biosynthesis in *S. cerevisiae* and its regulation by the TORC2-Ypk1 signaling axis. *CerS*, ceramide synthase. *D,* the levels of the LCB C18-dihydrosphingosine (*C18-DHS*) and C18-phytosphingosine (*C18-PHS*) and of ceramides (presented as the sum of all species) in lipid extracts from WT cells, *vps4*Δ and *vps4*Δ *ypk1*Δ mutants were measured using LC–MS and quantified using nonyeast LCBs/ceramides as external standards. Data are normalized to WT levels of C18-PHS and ceramide (set to 1) and presented as mean ± S.D. from 3 independent experiments. The *circles* indicate the individual measurements. *E,* autoradiogram of sphingolipid extracts from [^3^H]serine-radiolabeled WT cells and the indicated mutants that were separated by TLC. *X* indicates an unknown lipid species that is insensitive to myriocin treatment (data not shown). *F,* equal amounts of *vps4*Δ *ypk1*Δ cells expressing the indicated plasmids were spotted in serial dilutions on a YPD agar plate and incubated at 26 °C. See also Figs. S2 and S3.

Next, we analyzed how TORC2-Ypk1 signaling and the function of the ESCRT machinery converge to mitigate membrane stress. Therefore, we defined the molecular nature of membrane stress in ESCRT mutants that is controlled by TORC2-Ypk1 signaling. Ypk1 is activated by TORC2 through phosphorylation primarily of Ser-644 and Thr-662 (Fig. 1*C*) (13). Importantly, ESCRT mutants not only had slightly higher steady state Ypk1 protein levels, but also higher levels of active Ypk1 phosphorylated on Thr-662 (pThr-662) (Fig. 3*A*, Fig. S2, A and *B*). We found no evidence for ESCRT-dependent lysosomal turnover of Ypk1 (34) in growing cells (Fig. S2, *C* and *D*). The Ypk1 activation level in ESCRT mutants was substantial, but still lower compared with the hyperactivation of TORC2 signaling upon treatment with the SPT inhibitor myriocin (Fig. 3*A*, *lane 1*). Of note myriocin treatment for 150 min also reproducibly resulted in the up-regulation of Ypk1 protein levels (Fig. 3*A*, *lane 1*). The elevated Ypk1 protein levels that were detected in ESCRT mutants and in response to myriocin treatment might thus reflect Ypk1 stabilization by TORC2 phosphorylation as described earlier (35). We also observed a slight increase of *YPK1* mRNA in *vps4*Δ cells (Fig. S2*E*).

Ypk1 has a range of different cellular targets (18, 19, 21–24, 27). Among those, the paralogous ER proteins Orm1 and Orm2 are the essential targets (9, 18). Consistent with elevated TORC2-dependent activation of Ypk1 in ESCRT mutant cells, the fraction of phosphorylated Orm2 was increased (Fig. 3*B*, *lane 1*, Fig. S2*F*). Interestingly, also Orm2 protein levels were increased (Fig. 3*B*, *lane 1,* Fig. S2*F*). Deletion of *YPK1* reduced, but did not completely abrogate, the fraction of phosphorylated Orm2 (presumably due to the presence of Ypk2), and also caused up-regulation of Orm2 protein levels (Fig. 3*B*, *lane 3,* Fig. S2*F*). Also in *vps4*Δ *ypk1*Δ double mutants the fraction of phosphorylated Orm2 was reduced, whereas the protein levels of Orm2 were higher than in either a single mutant or in WT cells (Fig. 3*B*, *lane 4,* Fig. S2*F*). These results suggested that TORC2-Ypk1 signaling helped to keep Orm2 protein levels low in ESCRT mutants and WT cells. Moreover, it implied that the ESCRT machinery could somehow be involved in the regulation of Orm2.

### TORC2-Ypk1 signaling prevents detrimental SL imbalance in ESCRT mutants

TORC2-Ypk1–dependent phosphorylation of Orm2 and its paralogue Orm1 de-represses the synthesis of LCBs by the SPT complex (8, 9). TORC2 and Ypk1 also stimulate a subsequent step in the SL biosynthetic pathway by phosphorylating Lag1 and Lac1, the ER-resident ceramide synthases (CerS) (Fig. 3*C*). This concerted regulation avoids the toxic accumulation of biosynthetic intermediates and helps to maintain SL homeostasis (19).

The steady state levels of total LCBs (C18-PHS and C18-DHS) measured using liquid chromatography-MS increased ∼2-fold in *ypk1*Δ cells compared with WT cells, whereas ceramide levels did not change dramatically (Fig. S3*A*). In contrast, in *vps4*Δ cells, despite the accumulation of Orm2, total LCBs and ceramides were not substantially different from WT cells (Fig. 3*D*). Yet, the loss of Ypk1 in *vps4*Δ mutants skewed SL biosynthesis. In *vps4*Δ *ypk1*Δ double mutants, the steady state LCB levels were increased (>3-fold) beyond the level of *ypk1*Δ single mutants, whereas ceramide levels remained similar to WT cells or *vps4*Δ or *ypk1*Δ single mutants (Fig. 3*D*, Fig. S3*A*). It seemed that the loss of Ypk1 signaling in ESCRT mutants caused an imbalance of SPT and CerS activity, implying that the activity of CerS might have become a bottleneck.

Consistently, metabolic labeling of the SL pool with l-[^3^H]serine showed that *vps4*Δ *ypk1*Δ double mutants produced less complex SL (predominantly IPC, but also MIPC and M(IP)_2_C) compared with WT cells and *vps4*Δ or *ypk1*Δ mutants, which showed only subtle variations in the steady state levels of complex SL species (Fig. 3*E*, Fig. S3*B*). Expression of a phosphomimetic variant of CerS (*LAG1*-S23E,S24E) that promotes the formation of ceramides and complex SL independently of Ypk1 activity (19, 28) partially restored the growth of *vps4*Δ *ypk1*Δ cells (Fig. 3*F*).

Taken together, these results demonstrate that ESCRT mutants have elevated Orm2 protein levels and that TORC2-Ypk1 phosphorylation is required to compensate for the defects ensuing from Orm2 accumulation. Due to compensatory TORC2-Ypk1 signaling activity, the SL profile of ESCRT mutants shows only subtle differences compared with WT cells. However, loss of *YPK1* in ESCRT mutants caused a further increase in Orm2 proteins, which led to a detrimental imbalance in SPT and CerS activity characterized by an accumulation of LCBs and their impaired conversion into ceramides and complex SL. This imbalance in SL homeostasis might contribute to the defects in viability and PM integrity observed in *vps4*Δ *ypk1*Δ mutants.

### In ESCRT mutants Orm2 ER export is hampered and slows down Orm2 degradation via EGAD

Although Orm2 protein levels were up-regulated in ESCRT mutants, the protein levels of Orm1 or other subunits of the SPOTS complex were unaffected (Fig. S4*A*). The changes in *ORM2* mRNA levels were minor and similar to *ORM1* (Fig. S4*B*), and cannot explain the increase of Orm2 protein. We concluded that the increase in Orm2 protein levels was caused by selective post-transcriptional mechanisms. The protein levels of Orm2 (but not Orm1) are controlled by a multistep process resulting in its EGAD-mediated degradation (6). First, Orm2 is phosphorylated on Ser-46, -47, and -48 by Ypk1. Next, phosphorylated Orm2 is exported from the ER in a COP-II–dependent manner. Once Orm2 arrives at the Golgi and on endosomes, it is recognized and ubiquitinated by the Dsc ubiquitin ligase complex, extracted from the membrane by Cdc48, and finally degraded by proteasomes (6).

**Figure 4.**
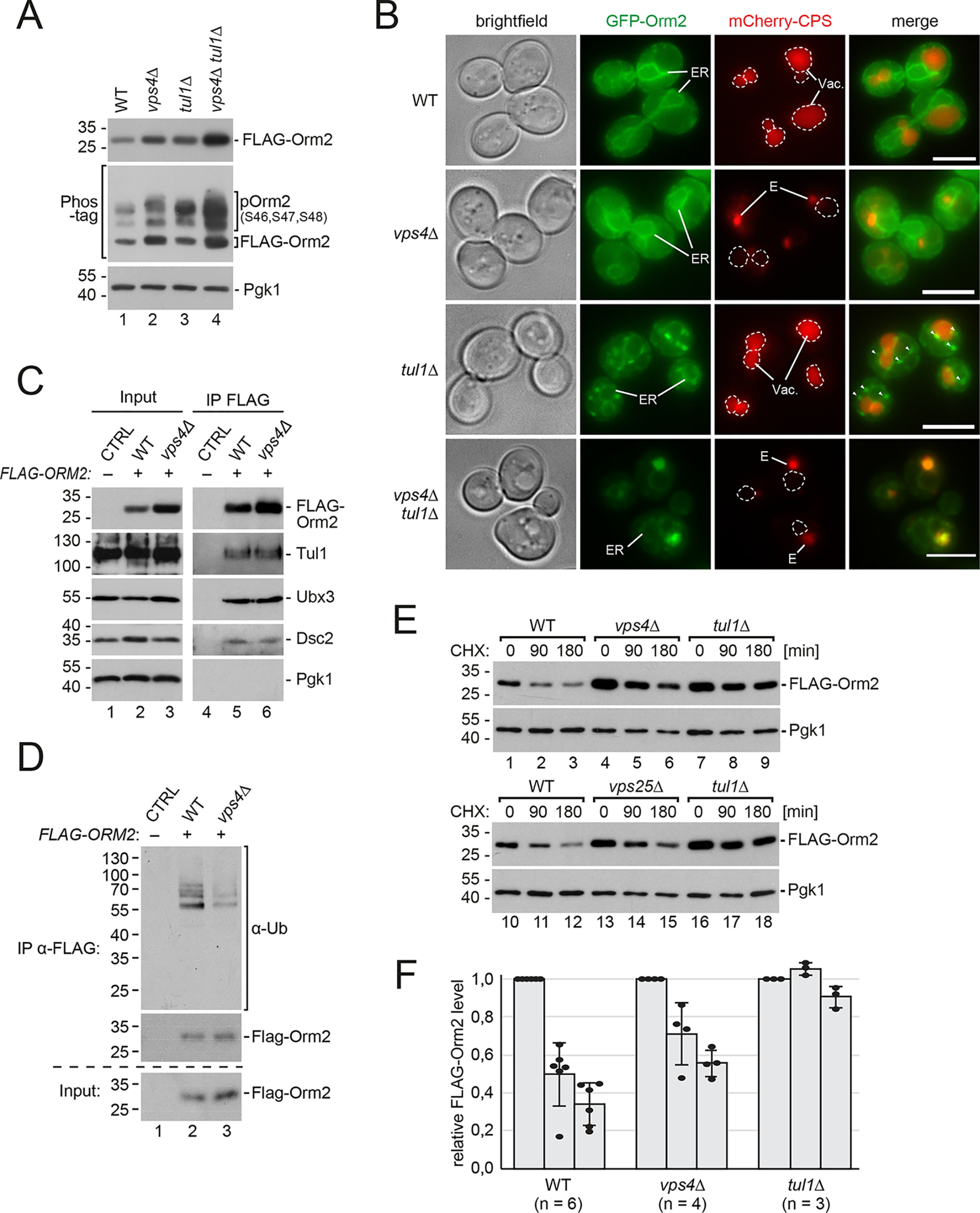
**In ESCRT mutants Orm2 ER export is hampered and slows down Orm2 degradation via EGAD.** *A,* SDS-PAGE, Phos-tag SDS-PAGE, and Western blotting analysis with the indicated antibodies of total yeast lysates from WT cells and the indicated mutants expressing *FLAG-ORM2*. *B,* epifluorescence and phase-contrast microscopy of living WT cells and the indicated mutants expressing GFP-Orm2 WT (*green*) and mCherry-CPS (*red*). *E*, class E compartment. *Dashed circles* indicate the position of vacuoles (*Vac*). *Arrowheads* indicate GFP-Orm2 accumulations in post-ER compartments. *Scale bars*, 5 μm. *C,* SDS-PAGE and Western blotting analysis with the indicated antibodies of input and elution (with FLAG peptide) from native FLAG-Orm2 IP from WT cells and *vps4*Δ mutants. Control cells expressed untagged Orm2. *D,* SDS-PAGE and Western blotting analysis with the indicated antibodies of input and elution (with FLAG peptide) from denaturing FLAG-Orm2 immunoprecipitations from WT cells and *vps4*Δ mutants. *E,* WT cells and the indicated mutants were left untreated (0 min) or were treated with CHX to block protein synthesis for 90 and 180 min at 26 °C. Total cell lysates were analyzed by SDS-PAGE and Western blotting with the indicated antibodies. *F,* graphs display the FLAG-Orm2 protein levels determined by densitometric quantification of Western blots from cell lysates of WT cells and the indicated mutants at 0, 90, and 180 min after the addition of CHX. Each experiment was repeated at least three times, FLAG-Orm2 levels were normalized to Pgk1 loading controls, and each time point was related to *t* = 0 min (set to 1). Data are presented as mean ± S.D. The *circles* indicate the individual measurements. *A–F*, all strains are in *orm2*Δ background. See also Fig. S4.

To understand which of these steps are affected in ESCRT mutants, we compared them to mutants of the Dsc complex (*tul1*Δ), which block EGAD. Orm2 protein levels and its phosphorylation were similar in ESCRT (*vps4*Δ) and EGAD (*tul1*Δ) mutants (Fig. 4*A*, *lanes 2* and *3*), but increased additionally in *vps4*Δ *tul1*Δ cells (Fig. 4*A*, *lane 4*). In *tul1*Δ mutants GFP-Orm2 accumulated at the ER and on post-ER compartments, including Golgi, endosomes, and the vacuolar limiting membrane, as reported earlier (6). In contrast, in *vps4*Δ cells, GFP-Orm2 was detected almost exclusively at the ER (Fig. 4*B*). Only in some cells, we observed a partial colocalization of GFP-Orm2 with the ESCRT substrate mCherry-CPS in class E compartments (Fig. S4*C*). Thus, Orm2 accumulated in different subcellular compartments in ESCRT and EGAD mutants.

Consistent with earlier reports (36), we detected Tul1 and Ubx3, two essential components of the Dsc complex, at the class E compartment in ESCRT mutants (Fig. S4*D*). Hence, the Dsc complex could degrade Orm2 once it reached the class E compartment. Indeed, GFP-Orm2 strongly accumulated in class E compartments when the ESCRT machinery and the EGAD pathway were compromised in *vps4*Δ *tul1*Δ cells (Fig. 4*B*), in agreement with our earlier findings (6). The mutant Orm2-K25R,K33R, which is no longer ubiquitinated by the Dsc complex and hence no longer degraded, was also found predominantly in class E compartments in ESCRT mutants (Fig. S4*E*).

To biochemically analyze if Orm2 was still an EGAD substrate in ESCRT mutants, we performed native immunoprecipitation (IP) of FLAG-Orm2 and found that a fraction of Orm2 still physically interacted with the Dsc complex in *vps4*Δ cells (Fig. 4*C*). Additionally, denaturing IP of FLAG-Orm2 showed that it was still ubiquitinated (Fig. 4*D*), although to a lower extent compared with WT cells. These results suggested that the Dsc complex still interacted with and ubiquitinated Orm2 in the post-ER compartments of ESCRT mutants. Consistently, cycloheximide chases demonstrated that Orm2 was still degraded in two different ESCRT mutants (*vps4*Δ and *vps25*Δ), although slower compared with WT cells (Fig. 4*E*). In WT cells Orm2 was degraded with a *t*_1/2_ of ∼90 min, and in *vps4*Δ mutants the *t*_1/2_ of Orm2 was ∼180 min (Fig. 4, *E* and *F*). In contrast, Orm2 degradation was fully blocked in mutants of the Dsc complex (*tul1*Δ) (6) (Fig. 4, *E* and *F*). Collectively, these results show an apparent accumulation of Orm2 at the ER of ESCRT mutants, indicating reduced ER export of Orm2 and hence a slower EGAD-dependent degradation.

### Enforcing ER export of Orm2 in ESCRT mutants leads to its EGAD-dependent degradation and relieves membrane stress

**Figure 5.**
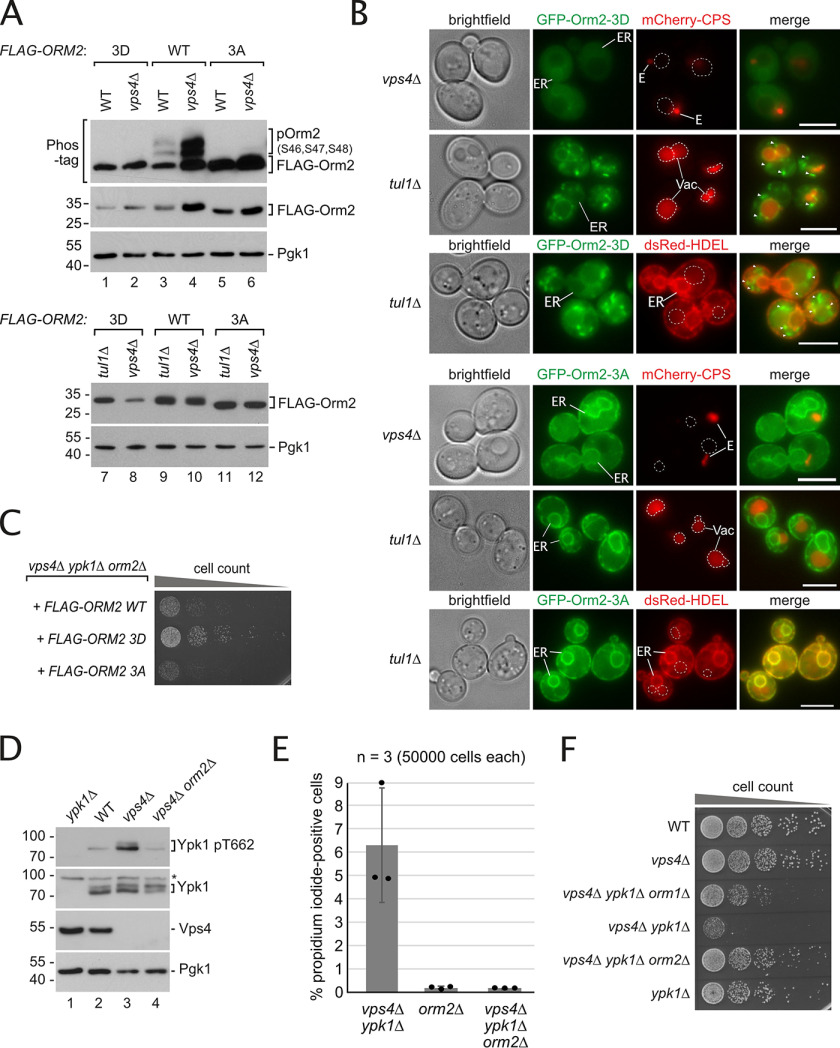
**Enforcing ER-export of Orm2 in ESCRT mutants leads to its EGAD-dependent degradation and relieves membrane stress.** *A,* SDS-PAGE or Phos-tag SDS-PAGE and Western blotting analysis with the indicated antibodies of total yeast lysates from WT, *vps4*Δ, or *tul1*Δ cells expressing *FLAG-ORM2-WT, -3A,* or *-3D* (in *orm2*Δ background). *B,* epifluorescence and phase-contrast microscopy of living *vps4*Δ or *tul1*Δ cells expressing GFP-Orm2-3D or -3A (*green*) and mCherry-CPS or the ER marker dsRed-HDEL (*red*). *E*, class E compartment. *Dashed circles* indicate the position of vacuoles (*Vac*). *Arrowheads* indicate GFP-Orm2 accumulations in post-ER compartments. *Scale bars*, 5 μm. *C,* equal amounts of *vps4*Δ *ypk1*Δ *orm2*Δ cells expressing the indicated *FLAG-ORM2* plasmids were spotted in serial dilutions on an auxotrophic selection medium agar plate and incubated at 26 °C. *D,* SDS-PAGE and Western blotting analysis with the indicated antibodies of total yeast lysates from WT cells and the indicated mutants. *E,* the indicated strains were grown into mid-log phase in YPD medium and stained with propidium iodide for 10 min and analyzed by FACS. Data are presented as mean ± S.D. from 3 independent experiments. The data for the *vps4*Δ *ypk1*Δ strain is the same as described in Fig. 2*E* and was plotted again for comparison. *F,* equal amounts of WT cells and indicated single, double, or triple mutants in serial dilutions were incubated on a YPD agar plate at 26 °C. See also Fig. S5.

Our results so far present a conundrum: in ESCRT mutants Orm2 was phosphorylated by TORC2-Ypk1 (Fig. 3*B*, Fig. 4*A*), but still accumulated at the ER. We reported earlier that Ser-46, -47, and -48 to aspartic acid mutations (Orm2-3D) mimic constitutive phosphorylation by Ypk1 and trigger constitutive ER export and constant degradation via EGAD. Consistently, Orm2-3D protein levels were low in WT cells (Fig. 5*A*, *lane 1*). Importantly, also in *vps4*Δ mutants Orm2-3D protein levels were low and comparable with WT cells (Fig. 5*A*, *lane 2*), whereas Orm2-3D protein levels remained high in EGAD (*tul1*Δ) mutants where Orm2-3D is not degraded (Fig. 5*A*, *lane 7*). Conversely, mutation of the Ypk1 phosphorylation sites in Orm2 to alanine (Orm2-3A) (9) strongly reduced Orm2 ER export and subsequent EGAD (6), and therefore Orm2-3A accumulated in WT cells, and *vps4*Δ and *tul1*Δ mutants alike (Fig. 5*A*, *lanes 5* and *6* and *11* and *12*).

These findings were corroborated using live cell fluorescence microscopy. In EGAD-deficient cells (*tul1*Δ), GFP-Orm2-3D was constitutively exported from the ER but not degraded, and therefore accumulated in post-ER compartments (Fig. 5*B*). In contrast, in *vps4*Δ as well as in *vps4*Δ *ypk1*Δ mutants, GFP-Orm2-3D no longer accumulated and was barely detected at the ER or in post-ER compartments (Fig. 5*B*, Fig. S5*A*). As expected, GFP-Orm2-3A accumulated at the ER in *vps4*Δ and *tul1*Δ cells (Fig. 5*B*). Remarkably, expression of the Orm2-3D mutant, but not Orm2-WT or Orm2-3A, improved the growth of *vps4*Δ *ypk1*Δ cells (Fig. 5*C*).

We therefore tested if accumulation of Orm2 at the ER constituted the major membrane stress factor in ESCRT mutants. In *vps4*Δ *orm2*Δ double mutants Ypk1 activation by TORC2 (pThr-662) was no longer elevated (Fig. 5*D*). In addition, *vps4*Δ *orm2*Δ cells grew better in the presence of PalmC than *vps4*Δ single mutants, suggesting a decreased susceptibility to PM stress (Fig. S5*B*). Furthermore, deletion of *ORM2* in *vps4*Δ *ypk1*Δ double mutants restored membrane integrity and decreased the fraction of PI-positive cells to *orm2*Δ single mutant levels (Fig. 5*E*). Moreover, the deletion of *ORM2* fully rescued the growth defect of *vps4*Δ *ypk1*Δ cells (Fig. 5*F*). A partial rescue was also observed upon deletion of *ORM1*.

Taken together, the deletion of Orm2 reduced TORC2 activation and improved PM integrity and viability of ESCRT mutants. In addition, mimicking the constitutive Ypk1-dependent phosphorylation of Orm2 restored ER export and the subsequent EGAD-dependent Orm2 degradation in ESCRT mutants, which was sufficient to improve the viability of *vps4*Δ *ypk1*Δ mutants. These results implied that, rather than general defects in ER export or in Orm2 degradation, untimely dephosphorylation caused the detrimental accumulation of Orm2 at the ER.

### Calcineurin counteracts the ER export of Orm2

The calcium-dependent protein phosphatase calcineurin is a major antagonist of the TORC2-Ypk1 signaling axis (19, 37), with regulatory roles in sphingolipid biosynthesis (16, 19, 28, 38). ESCRT mutant cells have been reported to be prone to accumulate intracellular calcium (39), with increased activity of a calcineurin-dependent transcriptional reporter (40). Therefore, we hypothesized that calcineurin activity in ESCRT mutants could counteract TORC2-Ypk1 signaling and thereby delay ER export of Orm2.

**Figure 6.**
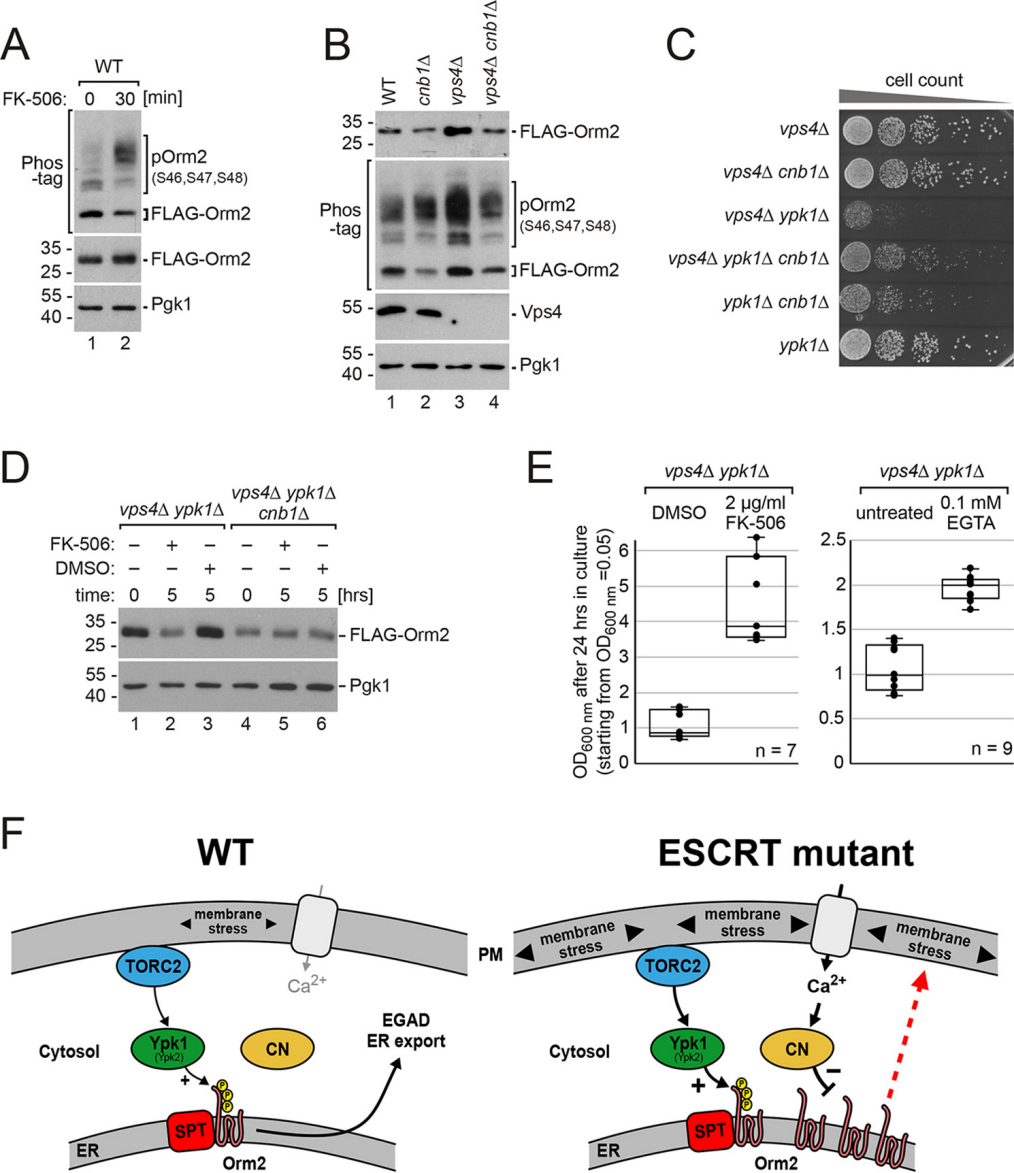
**Calcineurin counteracts the ER export of Orm2.** *A,* SDS-PAGE and Phos-tag SDS-PAGE and Western blotting analysis with the indicated antibodies of total yeast lysates from WT cells expressing *FLAG-ORM2* (in *orm2*Δ background) treated with FK-506 (2 µg/ml) for the indicated time. *B,* SDS-PAGE and Phos-tag SDS-PAGE and Western blotting analysis with the indicated antibodies of total yeast lysates from WT cells and the indicated mutants expressing *FLAG- ORM2*. Quantification is shown in Fig. S6*A*. *C,* equal amounts of the indicated single, double, or triple mutants in serial dilutions were incubated on a YPD agar plate at 26 °C. *D, left*: growth of *vps4*Δ *ypk1*Δ cells in YPD medium in presence of 2 µg/ml of FK-506 or vehicle (DMSO). *Right*: growth of *vps4*Δ *ypk1*Δ cells in YPD medium in the presence of 0.1 mm EGTA or untreated. Cells were inoculated to OD_600 nm_ = 0.05 and grown for 24 h in 3 independent experiments. The *circles* indicate the individual measurements (7 and 9 technical replicates). *E,* SDS-PAGE PAGE and Western blotting analysis with the indicated antibodies of total yeast lysates from *vps4*Δ *ypk1*Δ and *vps4*Δ *ypk1*Δ *cnb1*Δ cells expressing *FLAG-ORM2* treated with FK-506 (2 µg/ml) or vehicle (DMSO) for the indicated time. *F,* model for the post-translational regulation of Orm2 protein levels in WT cells and its deregulation in ESCRT mutants. *CN*, calcineurin. See also Fig. S6.

To test this hypothesis, we acutely inhibited calcineurin with FK-506. It had been shown before that calcineurin inhibition does not augment Ypk1 activation by TORC2 or Pkh1/2 (9, 14, 19). Nevertheless, inhibition of calcineurin for 30 min resulted in hyperphosphorylation of Orm2 (Fig. 6*A*). Next, we deleted the regulatory calcineurin subunit Cnb1, which chronically abrogates calcineurin activity (41). Chronic loss of calcineurin activity led to a decrease in Orm2 protein levels and a concomitant increase in the fraction of phosphorylated Orm2 in *cnb1*Δ cells compared with WT cells (Fig. 6*B*, compare *lanes 1* and *2*, Fig. S6*A*). This decrease of Orm2 protein levels was mediated by EGAD, because it required the lysine residues in Orm2 (Lys-25, Lys-33) that are ubiquitinated by the Dsc complex (6). In *cnb1*Δ cells, the protein levels of Orm2-K25R,K33R were no longer reduced compared with WT cells, although Orm2-K25R,K33R was still hyperphosphorylated (Fig. S6*B*).

In *vps4*Δ mutants, deletion of *CNB1* decreased the accumulation of Orm2 to protein levels that were now similar to WT cells, and also caused an increase in the fraction of phosphorylated Orm2 (Fig. 6*B*, compare *lanes* 3 and *4*, Fig. S6*A*). In *cnb1*Δ and *vps4*Δ *cnb1*Δ mutants the remaining GFP-Orm2 signal localized to the ER (Fig. S6*C*). It seemed that calcineurin either directly or indirectly led to the dephosphorylation of Orm2, thereby antagonized TORC2-Ypk1–induced ER export of Orm2 and thus counteracted its degradation.

Moreover, this calcineurin activity also rendered ESCRT mutants dependent on TORC2-Ypk1 signaling. Deletion of *CNB1* partially restored the growth of *vps4*Δ *ypk1*Δ mutants (Fig. 6*C*), reduced Orm2 protein levels (Fig. 6*D*, compare *lanes* 1 and *4*), and attenuated the accumulation of GFP-Orm2 at the ER (Fig. S6*C*). Likewise, pharmacological inhibition of calcineurin (2 µg/ml of FK-506) markedly decreased Orm2 protein levels in the *vps4*Δ *ypk1*Δ double mutants (Fig. 6*D*). FK-506 treatment or addition of the calcium-chelator EGTA to the medium also improved the growth of *vps4*Δ *ypk1*Δ cells (Fig. 6*E*). The effects of FK-506 and EGTA were dependent on calcineurin, because additive effects could not be observed once *CNB1* was disrupted (Fig. 6, *D* and *E*, Fig. S6*D*). The transcription factor Crz1 is a major downstream effector of calcineurin and also promotes ER stress-induced transcription of *ORM2* (42, 43). Yet, unlike the deletion of *CNB1*, deletion of *CRZ1* did not rescue growth of *vps4*Δ *ypk1*Δ cells (Fig. S6*D*). This is consistent with a role of calcineurin in controlling either directly or indirectly Orm2 phosphorylation.

We conclude that calcineurin activity hampered the ER export of Orm2 and its subsequent EGAD-dependent degradation. The ensuing accumulation of Orm2 at the ER triggered membrane stress and rendered the growth of ESCRT mutants dependent on TORC2-Ypk1 signaling to prevent imbalance in SL homeostasis and loss of plasma membrane integrity (Fig. 6*F*).

## Discussion

Here we show that the ESCRT machinery contributes to PM homeostasis in budding yeast and takes part in ER-PM cross-talk to preserve the barrier function of the PM. Our work connects the ESCRT machinery to PM stress and deregulated calcineurin signaling, and explains how ESCRT mutants become addicted to TORC2-dependent signaling (Fig. 6*F*).

We observed the formation ESCRT assemblies at the PM in response to indirect (by reduction of membrane tension) and direct TORC2 inhibition. The major ESCRT-III subunit Snf7 and the AAA-ATPase Vps4 were detected at PM invaginations and subcortical foci, which are reminiscent of stalled endocytic buds. This seems plausible, because TORC2 promotes endocytosis by controlling actin and endocytic proteins and by modulating membrane tension (18, 20, 23). In human cells, ESCRT complexes can repair damaged PM patches and also prolong PM integrity during necroptosis (44–47). There, injury-triggered calcium increase results in assembly of ESCRT-III and accessory proteins at the site of repair via Alix (47). Importantly, we did not observe obvious membrane lesions near ESCRT-III foci. Rather than repairing the PM, we suggest that these ESCRT-III/Vps4 assemblies play a role in maintaining membrane integrity to prevent damage.

How ESCRT-III is recruited to the stressed (but probably still uninjured) regions of the PM in budding yeast is currently unclear, but several scenarios appear possible. (i) Changes in PM tension itself might support the recruitment of the ESCRT machinery (48, 49). (ii) ESCRT-III/Vps4 recruitment could be driven by ESCRT-0, -I, or -II complexes interacting with the ubiquitinated membrane proteins in these stalling endocytic buds. Alternatively, Bro1, similar to Alix in human cells, might help to recruit ESCRT-III directly to the PM. (iii) In budding yeast, the regulator of Ime2 (RIM) pathway recruits ESCRT-III subunits (Snf7) to the PM during adaptation to alkaline pH (50).

At the PM, the ESCRT machinery could consolidate a slack PM. This might be achieved either through stabilization of PM deformations, or via active membrane remodeling, which could increase membrane tension again. How exactly ESCRT-III/Vps4 assemblies help to maintain the integrity of the PM in response to tensile stress and TORC2 inactivation remains unclear at the moment.

It is clear, however, that the survival of ESCRT mutants required TORC2-Ypk1 signaling to maintain PM integrity. This is due to the accumulation of Orm2 at the ER, which emerges as the central agent for plasma membrane stress in ESCRT mutants. The ER accumulation of Orm2 is caused by its calcineurin-dependent dephosphorylation. Dephosphorylated Orm2 cannot efficiently exit the ER and hence its subsequent turnover through EGAD is hampered. This built-up of Orm2 at the ER perturbs the coordination of SPT and CerS activity in *vps4*Δ cells and must be counteracted through increased TORC2-Ypk1 activity (Fig. 6*F*). TORC2-Ypk1 signaling ensures SL homeostasis and matches LCB synthesis with ceramide synthesis. By phosphorylating Orm1/2 it de-represses SPT activity and by phosphorylating Lag1 and Lac1 it stimulates CerS activity (19). Thereby TORC2-Ypk1 signaling stimulates in a coordinated manner the flux of LCBs into ceramides and into complex SLs and prevents the potentially toxic imbalance of metabolic intermediates, such as LCBs and ceramides (38, 51). As long as this is possible, ESCRT mutants have little fitness defects. However, if phosphorylation of Orm2 by Ypk1 is additionally impaired (*e.g.* in *vps4*Δ *ypk1*Δ double mutants) this compensation cannot be maintained. Ypk2 can still de-repress SPT activity, despite the accumulation of Orm2. Yet, this mandates that most Ypk2 activity diverges away from other targets such as CerS. This is likely, because high Orm2 levels can also diverge Ypk1 (and hence probably also Ypk2) activity away from other important targets (19). As a result, LCBs are still synthesized, whereas CerS activity will not be stimulated, and hence LCB are no longer consumed. This ultimately leads to a detrimental imbalance of SL metabolites. The PM integrity becomes compromised, and cell viability of *vps4*Δ *ypk1*Δ mutants decreases dramatically. Consistent with Orm2 accumulation at the ER as a major membrane stressor, eliminating Orm2 genetically, forcing Orm2 ER export (either by expression of a phosphomimetic Orm2-3D mutant or by inhibition of calcineurin) and thereby stimulating EGAD-dependent degradation, or mimicking phosphorylation of CerS, improved the growth of *vps4*Δ *ypk1*Δ mutants.

Pharmacological inhibition of calcineurin or genetic disruption of calcineurin (*CNB1*) increased Orm2 phosphorylation and promoted efficient ER export of Orm2 and degradation. Moreover, the growth defect of *vps4*Δ *ypk1*Δ double mutants was alleviated by calcineurin inhibition and extracellular calcium chelation, suggesting that increased calcium uptake drives detrimental calcineurin activity in these cells. Calcineurin activity was found to be increased in ESCRT mutants earlier (39, 40). Increased calcium influx in ESCRT mutants may be caused by deregulation of the plasma membrane calcium channel (Cch1/Mid1), which is likely a substrate for ESCRT-dependent protein degradation. Mid1 could also respond to increased membrane stress in ESCRT mutants, because Mid1 is a stretch-activated channel and allows calcium influx and calcineurin signaling to respond to mechanical membrane stress (52). Alternatively, ER-PM contact sites may be corrupted in ESCRT mutants (53). The disruption of these contact sites leads to increased calcium influx and calcineurin activity, which increased LCBs and decreased ceramide levels (28). Remarkably, *vps4*Δ mutants up-regulate the Golgi calcium pump Pmr1 (54), possibly to decrease cytosolic calcium. Pmr1 activity appeared also essential in ESCRT mutants because we found in our screen that loss of *PMR1* impaired the survival of *vps4*Δ mutants (6).

Calcineurin antagonizes TORC2 and Ypk1 activity also toward many targets (17, 19, 37). If Orm2 is a direct substrate of calcineurin is not yet clear. However, in addition to lowering Orm2 protein levels (by promoting ER export and EGAD-dependent degradation), inhibition of calcineurin is also expected to promote phosphorylation of the CerS subunits Lag1/Lac1 and thereby formation of ceramides, which mitigates the apparent bottleneck in SL synthesis (19). Thus, calcineurin inactivation probably has additional beneficial effects in ESCRT mutants, which collectively render their growth largely independent of Ypk1 signaling.

In summary, we propose that ESCRT function maintains PM integrity on several levels (see Fig. 6*F*): (i) through the MVB pathway by degrading membrane proteins and preventing proteotoxic stress (55), (ii) by directly preserving and/or repairing the PM in response to membrane stresses including TORC2 inactivation, and (iii) by maintaining calcium homeostasis, which is important to restrain the activity of phosphatase calcineurin. In particular, calcineurin works against TORC2- and Ypk1-dependent phosphorylation of Orm2, and thereby decreases its ER export and EGAD-dependent degradation. Upon membrane stress induced by loss of ESCRT function, increased TORC2/Ypk1 signaling counteracts calcineurin-dependent Orm2 accumulation and thereby ensures coordinated biosynthetic SL flux and PM homeostasis. As such, the ESCRT, TORC2/Ypk1, and EGAD pathways share interconnected and compensatory roles to maintain the integrity of the PM.

## Experimental procedures

### Yeast strains, plasmids, and growth conditions

Genetic modifications were performed by PCR and/or homologous recombination using standard techniques. Plasmid-expressed genes including their native promoters and terminators were amplified from yeast genomic DNA and cloned into centromeric vectors (pRS series). Tagged version of Orm1 and Orm2 (3xHA, 3xFLAG, or GFP) and their respective mutants were expressed from plasmids in the respective deletion mutants replacing the endogenous protein (with exception of Fig. 5*D*, where *FLAG-Orm2* was co-expressed). All constructs were analyzed by DNA sequencing and transformed into yeast cells using standard techniques. Genotypes of yeast strains and plasmids used in this study as well as primers for PCR-based genetic modifications and cloning are listed in Table S2.

All *S. cerevisiae* strains in this study were SEY6210 derivatives. For liquid cultures, cells were incubated in YNB synthetic medium supplemented with amino acids (according to respective auxotrophies) and 2% glucose at 26 °C in a shaker and grown to midlog phase (OD_600_ = 0.5–0.8). For growth on agar plates, yeast cells were diluted to OD_600 nm_ = 0.05 and spotted in serial dilutions on YPD or YNB (auxotrophic selection medium) plates at the indicated conditions. Rapamycin (Sigma) was added at the indicated concentrations from a 1 mm stock in DMSO and myriocin (Sigma) was added to 1.5 μm from a 5 mm stock in methanol. Untreated controls were supplied with the appropriate amount of the respective solvent. For the assessment of growth in the presence of PalmC, cells were grown into log phase, diluted to an OD_600 nm_ of 0.1 into fresh YNB medium containing PalmC (Sigma) at the indicated concentration (from a 5 mm stock in DMSO), and grown at 26 °C, 180 rpm. For the assessment of growth in the presence of FK-506 (Sigma; stock 20 mg/ml in DMSO) or EGTA (Sigma; stock 500 mm in water) cells were grown into log phase, diluted to an OD_600 nm_ of 0.05 into fresh YPD medium containing 2 µg/ml of FK-506 or 0.1 mm EGTA. After 24 h the OD_600 nm_ was measured.

### Analysis of genetic interaction data

GO enrichment analysis was performed using the 119 genes listed in appendix Table S1 of Ref. 6, as synthetically lethal with *vps4*Δ in two biological replicates or lethal in one and sick in the other replicate. They were analyzed with the “generic GO-Slim: process” terms using the GO-Slim mapper of the *Saccharomyces* genome database. GO term fusion and enrichment analysis was performed as described (6). Only significantly scored GO terms (*p* < 0.05) are presented in Fig. 1*A*. The full analysis is presented in Table S1.

### Preparation of whole cell protein extracts, Western blotting analysis, and immunodetection

To prepare whole cell lysates, proteins were extracted by post-alkaline lysis. When protein phosphorylation was also analyzed, extraction was done by a modified protocol with phosphatase inhibition as described (6). Protein extracts were denatured in Laemmli sample buffer, separated by SDS-PAGE (Bio-Rad Mini Protean), and transferred to polyvinylidene difluoride membranes by semi-dry electroblotting. Phos-tag SDS-PAGE as well as immunodetection of ubiquitinated Orm2 were done as described (6). Antibodies used in this study are listed in Table S2.

### FLAG-Orm2 immunoprecipitations

Immunoprecipitations of FLAG-Orm2 under denaturing or nondenaturing lysis conditions were done as described in detail in Ref. 6.

### RNA isolation and quantitative PCR (RT-qPCR)

RNA isolation, cDNA synthesis, and quantitative RT-PCR analysis were performed as described previously (6). TaqMan gene expression assays were from Thermo Fisher (*YPK1,* Sc04141261_s1; *ORM1,* Sc04125000_s1; *ORM2,* Sc04149509_s1; housekeeping gene *PGK1,* Sc04104844_s1). RT-qPCR analysis was done from 4 to 6 independent biological samples and each in 3-4 technical replicates. Data were analyzed with the PikoReal software (version 2.2; Thermo Scientific) with manual threshold adjustment, and relative mRNA abundance was calculated in Microsoft Excel (Version 16.16.2; RRID:SCR_016137) using the ΔΔ*C_T_* method.

### Fluorescence live cell wide field microscopy

For microscopy, cells were grown to midlog (OD_600_ = 0.5–0.8) phase in YNB media, concentrated by centrifugation, and directly mounted onto glass slides. For imaging of the Dsc complex (Fig. S4*B*), cells were grown for 36 h on selective YNB agar plates, dissolved in sterile water and mounted for imaging. Live cell wide field fluorescence microscopy was carried out using a Zeiss Axio Imager M1 equipped with a sola light engine LED light source (Lumencore), a ×100 oil immersion objective (NA 1.45) standard GFP, and mCherry fluorescent filters, a SPOT Xplorer CCD camera, and Visitron VisiView software (version 2.1.4). The brightness and contrast of the images in the figures were adjusted using Photoshop CS5 (Adobe Version 12.0.4 × 64; RRID:SCR_014199). For merged images the levels of red and green channels were separately adjusted.

### Assessment of plasma membrane integrity by propidium iodide staining

Cells were grown in YPD medium and kept in logarithmic growth phase for >12 h. 1 ml of culture was supplemented with 3 µg/ml of propidium iodide (Sigma; stock 1 mg/ml in PBS) and incubated at room temperature for 10 min. Cells were recovered by centrifugation (10,000 × *g*, 4 °C, 2 min), washed once with 1 ml of ice-cold PBS, resuspended in 0.5 ml of ice-cold PBS, and kept on ice. Propidium iodide-positive cells were counted on an Attune^TM^ NxT Acoustic Focusing Cytometer (Life Technologies) with Attune^TM^ NxT software (version 3.1.1243.0). Debris and cell doublets were excluded. Propidium iodide signal was measured with 488 nm excitation and emission in the 695/40 nm window. In each experiment 50,000 cells of each genotype were counted. Gating was adjusted so that more than 99.9% of WT cells were propidium iodide negative.

### Immunogold EM

As previously described (4) samples were high-pressure frozen, freeze-substituted and rehydrated (56), followed by indirect immunogold labeling of 100-nm thick, thawed cryosections (4). Antibodies used were goat polyclonal anti-GFP (1:500; Rockland) and rabbit anti-goat Fab' NANOGOLD™ (1:150; Nanoprobes). Transmission EM was performed on a Philips CM120 (now Thermo Fisher Scientific).

### Cycloheximide chase assay

Logarithmically growing cells (20 OD_600 nm_) were harvested by centrifugation and cells were resuspended in fresh medium to concentration of 0.4 OD/ml. 10 ml (4 OD_600 nm_) were immediately (*t* = 0 min) harvested by centrifugation, washed once with ice-cold 10 mm NaF solution, and pellets were snap frozen in liquid nitrogen. To the remaining culture 50 µg/ml of cycloheximide (Sigma Aldrich) was added from a 10 mg/ml stock. After the indicated time points 10 ml of culture were harvested, washed, and frozen as above. Whole cell extracts were prepared by alkaline extraction. SDS-PAGE, Western blotting detection, and quantification were done as described (6).

### Metabolic sphingolipid labeling, sphingolipid extraction, and TLC

Sphingolipid labeling and extraction were done as described previously (6, 57). Logarithmically growing cells (5 OD_600 nm_) were harvested by centrifugation and resuspended in 470 µl of fresh medium. 30 µCi of l-[^3^H]serine (1 µCi/µl; Hartmann Analytic) was added and cells were incubated at 30 °C, at 700 rpm for 5 h. Proteins were precipitated with 4.5% perchloric acid, cell pellets were washed with ice-cold 100 mm EDTA, resuspended in 50 µl of water, and subjected to mild alkaline methanolysis (50% methanol, 10% 1-butanol, 10% monomethylamine; 50 min at 50 °C) of ester lipids. Lysates were vacuum dried in a SpeedVac (37 °C), pellets were resuspended thoroughly in 300 µl of water by sonication, and tritium incorporation was assessed for normalization in duplicates by scintillation counting (LS6500, Beckmann Coulter). Sphingolipids were extracted 3 times with water-saturated 1-butanol, vacuum dried in a SpeedVac (37 °C), and dissolved in 50 µl of chloroform:methanol:water, 10:10:3, for TLC. TLC (solvent system chloroform, methanol, 4.2 n ammonium hydroxide, 9:7:2) was done as described (57) on aluminum silica TLC plates (Sigma) for 75 min. Subsequently, plates were dried under air flow and treated twice with En^3^Hance autoradiography enhancer (PerkinElmer Life Sciences), dried again, and exposed to autoradiography films (CL-Xposure Film, Thermo) at −80 °C. The identity of labeled bands was confirmed with specific mutant yeast strains or inhibitors (data not shown). A tritium-labeled band X close to the solvent front that was observed previously (57) is not sensitive to myriocin treatment (Sigma Aldrich, 1.5 μm), suggesting that it is not a sphingolipid.

### Lipid extraction and liquid chromatography-MS (LC–MS)

For combined LCB and ceramide analysis (Fig. 3*D*) lipids were extracted from lysed yeast cells according to 200 µg of protein by chloroform:methanol extraction (58). Prior to extraction an internal standard mix containing sphingosine (LCB, 17:0) and ceramide (CER, 18:0/17:1) was spiked into each sample for normalization. Quantification was done by external quantification with the same lipids in different concentrations. Dried lipid samples were dissolved in a 65:35 mixture of mobile phase A (60:40, water:acetonitrile, including 10 mm ammonium formate and 0.1% formic acid) and mobile phase B (88:10:2, 2-propanol:acetonitrile:H_2_O, including 2 mm ammonium formate and 0.02% formic acid). HPLC analysis was performed employing a C30 reverse-phase column (Thermo Acclaim C30, 2.1 × 250 mm, 3 μm, operated at 40° C; Thermo Fisher Scientific) connected to an HP 1100 series HPLC (Agilent) HPLC system and a QExactive*PLUS* orbitrap mass spectrometer (Thermo Fisher Scientific) equipped with a heated electrospray ionization probe. The elution was performed with a gradient of 20 min; from 0 to 1 min elution starts with 40% B and increases to 100% in a linear gradient over 13 min. 100% B is maintained for 3 min. Afterward solvent B was decreased to 40% and maintained for another 3.8 min for column re-equilibration. The mass spectrometer was run in negative and positive ion mode. The scan rate was set from 200 to 1200 *m*/*z*. Mass resolution was 70,000 with an AGC target of 3,000,000 and a maximum injection time of 100 ms. The MS was operated in data-dependent mode. For MS/MS the resolution was 35,000 with a maximum injection time of 50 ms and an AGC target of 100,000. The loop count was 10. Selected ions were fragmented by HCD (higher energy collision dissociation) with a normalized collision energy of 30. The dynamic exclusion list was set to 10 s to avoid repetitive sequencing. Ceramide peaks were identified using the Lipid Search algorithm (MKI, Tokyo, Japan). Peaks were defined through raw files, product ion, and precursor ion accurate masses. Ceramides were identified by database (>1,000,000 entries) search of negative and positive ion adducts. LCBs were identified by positive ion adducts. The accurate mass extracted ion chromatograms were integrated for each identified lipid precursor and peak areas obtained for quantitation. Internal standards were used for normalization and an external standard curve was used to calculate absolute values (in pmol/µg of protein). Ceramides are presented as the sum of all quantified ceramide species. For comparison, data were normalized to WT levels (set to 1) of ceramide and C18-PHS, respectively. Data are presented as mean ± S.D. from three independent experiments.

### Quantification and statistical analysis

Statistical details and sample numbers of quantitative analyses can be found in the respective figures and corresponding figure legends. Quantitative data are usually displayed as mean ± S.D. from at least 3 biological replicates and relates to the WT control.

#### 

##### GO analysis of genetic interaction data

A hypergeometric test was used to estimate if the mapped GO term is significantly enriched with the selected genes. The null hypothesis is that the selected genes are randomly sampled from all yeast genes. The resulting *p* values were corrected with the Benjamini-Hochberg method. All adjusted *p* values below 0.05 were reported.

##### Quantification of Snf7-eGFP and Vps4-eGFP localization

Images were taken in an unbiased manner by selecting and focusing cells only in brightfield view. 50 cells of each genotype and condition were imaged and visually inspected for the presence and number of eGFP puncta at the cell cortex. Cells were grouped into cohorts of 0-2, 3-5, and more than 5 cortical eGFP puncta.

##### Quantification of cell growth

Cell growth after 2 h in culture was measured photometrically by absorbance at 600 nm. Measurements from 3 independent experiments (each with up to three parallel cultures representing technical replicates) were presented with box-whisker plots indicating the median, first, and third quartile. Individual data points including outliers were also plotted.

##### Quantification of Western blotting analysis

Western blotting signals were quantified by densitometry using ImageJ2 (version 2.0.0-rc49/1.51h; RRID:SCR_003070) (59), quantifications were exported to Microsoft Excel (version 16.16.2; RRID:SCR_016137), normalized to the respective Pgk1 loading controls, and presented as mean ± S.D. from at least three independent experiments, *t* = 0 was set to 1. For the quantification of Phos-tag Western blotting signals, the intensities of unphosphorylated (lowest FLAG-Orm2 band) and phosphorylated (shifted bands) forms were quantified from the same exposure. Data were reported as percentage of the total FLAG-Orm2 signal (sum of phosphorylated and unphosphorylated forms). Due to technical reasons, phosphorylated proteins often transfer less efficiently in electroblots from Phos-tag gels (60). Therefore, the fraction of phosphorylated FLAG-Orm2 may be underestimated.

##### Quantitative PCR (RT-qPCR)

Mean, normalized ΔΔ*C_T_* values were individually calculated for four independent biological replicates (each with 3-4 technical replicates), log_2_-transformed to calculate fold-change, and presented as mean fold-change over WT ± S.D. A one-sided Student's *t* test was used on the mean ΔΔ*C_T_* values (*i.e.* prior to log transformation) of the four biological replicates to assess statistical significance.

##### Lipid extraction and MS

Internal standards for ceramides (Cer 18:1;2/17:0;0) and LCBs (LCB 17:0) spiked in prior to extraction were used for normalization and an external standard curve was used to calculate absolute values (in pmol/µg of protein). To compare data from different experimental setups, data were normalized to WT levels of the most abundant LCB and ceramide species (C18-phytosphingosine and Cer44:0;4). Data are presented as mean ± S.D. from three independent experiments.

## Data availability

All relevant data has been included in the paper in main figures and supplemental information.

## Supplementary Material

Supporting Information
