# Supplementary material for: TOR complex 2 (TORC2) signaling and the ESCRT machinery cooperate in the protection of plasma membrane integrity in yeast
Source: J Biol Chem. 2020 Jul 1;295(34):12028–44. doi: 10.1074/jbc.RA120.013222 (PMC7443507; doi:10.1074/jbc.RA120.013222)
Supplement: Supporting Information [file supp_295_34_12028__index.html]

TOR complex 2 (TORC2) signaling and the ESCRT machinery cooperate in the protection of plasma membrane integrity in yeast — The ESCRT machinery and membrane homeostasis — TOR complex 2 (TORC2) signaling and the ESCRT machinery cooperate in the protection of plasma membrane integrity in yeast — The ESCRT machinery and membrane homeostasis — Supporting Information 

# TOR complex 2 (TORC2) signaling and the ESCRT machinery cooperate in the protection of plasma membrane integrity in yeast

## Supporting Information

- Supporting Information for Schmidt et al - contains 6 supporting figures and 2 tables
