# Supplementary material for: TOR complex 2 (TORC2) signaling and the ESCRT machinery cooperate in the protection of plasma membrane integrity in yeast
Source: J Biol Chem. 2020 Jul 1;295(34):12028–44. doi: 10.1074/jbc.RA120.013222 (PMC7443507; doi:10.1074/jbc.RA120.013222)
Supplement: Supporting Information [file supp_RA120.013222_159103_2_supp_557998_qcsgl6.pdf]

Supporting information for:

TOR complex 2 (TORC2) signaling and the ESCRT machinery cooperate in the protection of plasma membrane integrity in yeast

Oliver Schmidt[1*], Yannick Weyer[1], Simon Sprenger[1], Michael A. Widerin[1], Sebastian Eising[2], Verena Baumann[1,3], Mihaela Angelova[4], Robbie Loewith[5], Christopher J. Stefan[6], Michael W. Hess[7], Florian Fröhlich[2], and David Teis[1]

[1] Institute for Cell Biology, Biocenter, Medical University of Innsbruck, Austria
[2] Department of Biology/Chemistry, University of Osnabrück, Germany
[3] current address: Max Perutz Labs, University of Vienna, Austria
[4] Cancer Evolution and Genome Instability Laboratory, Francis Crick Institute, London, UK
[5] Department of Molecular Biology, University of Geneva, Switzerland
[6] MRC Laboratory for Molecular Cell Biology, University College London, London, UK
[7] Institute for Histology and Embryology, Medical University of Innsbruck, Austria

*Corresponding author:
Dr. Oliver Schmidt
Medical University of Innsbruck, Biocenter, Division Cell Biology
Innrain 80/82, CCB Building
A-6020, Innsbruck, Austria

oliver.schmidt@i-med.ac.at
phone: +43 512 9003 70191
fax: +43 512 9003 73100

Content:

- Supporting information figures S1 – S6
- Supporting information tables S1 and S2

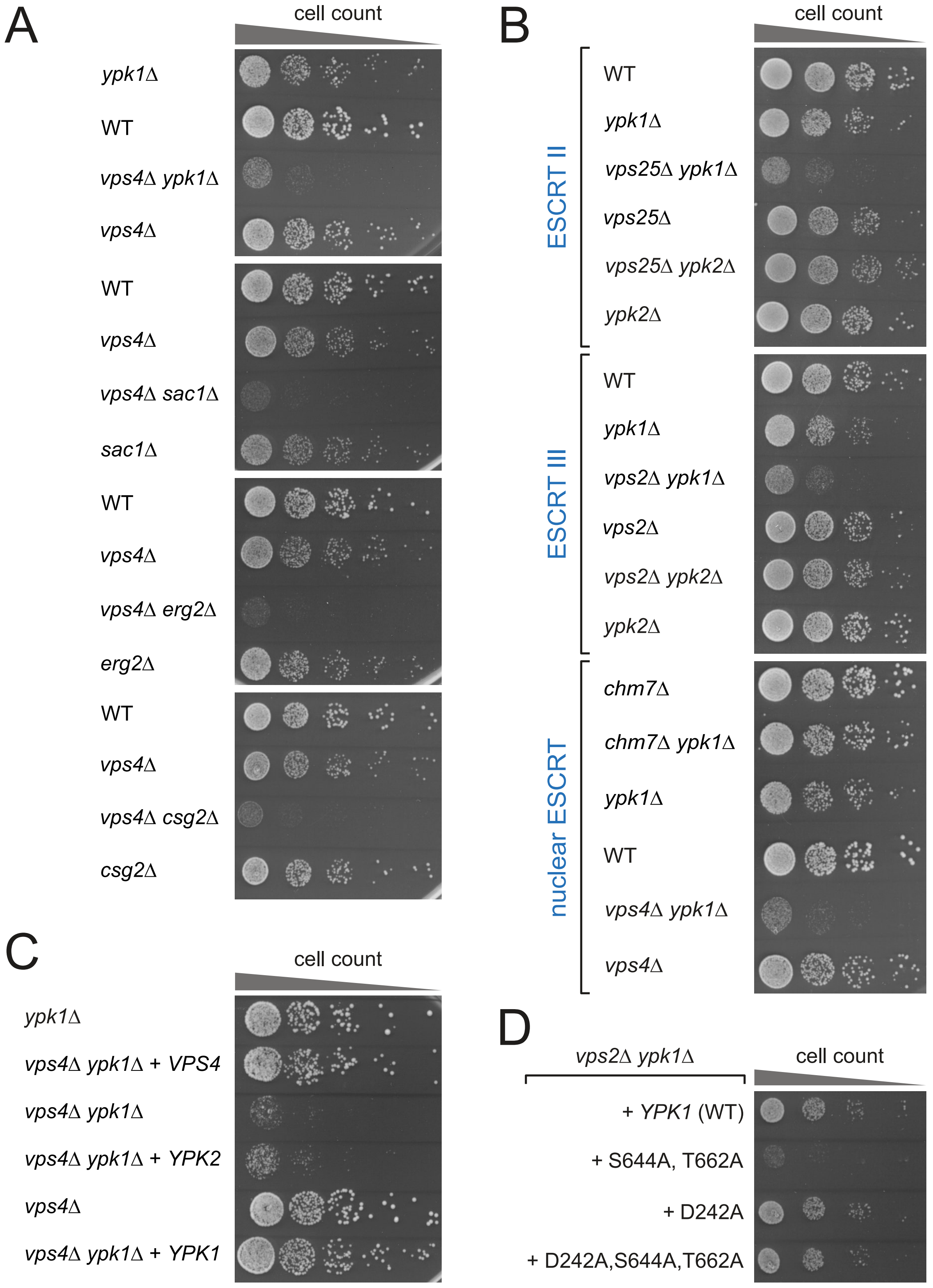

Figure S1 - related to Figure 1

Figure S1 - related to Figure 1.

(A, B) Equal amounts of WT cells and indicated single or double mutants in serial dilutions were incubated on auxotrophic selection medium agar plates at 26°C.

(C) Equal amounts of the indicated single or double mutants complemented with the indicated plasmids (or empty vectors for isogenic controls) in serial dilutions were incubated on auxotrophic selection medium agar plates at 26°C.

(D) Equal amounts of *vps2*Δ *ypk1*Δ double mutants expressing the indicated *YPK1* mutant plasmids in serial dilutions were incubated on auxotrophic selection medium agar plates at 26°C.

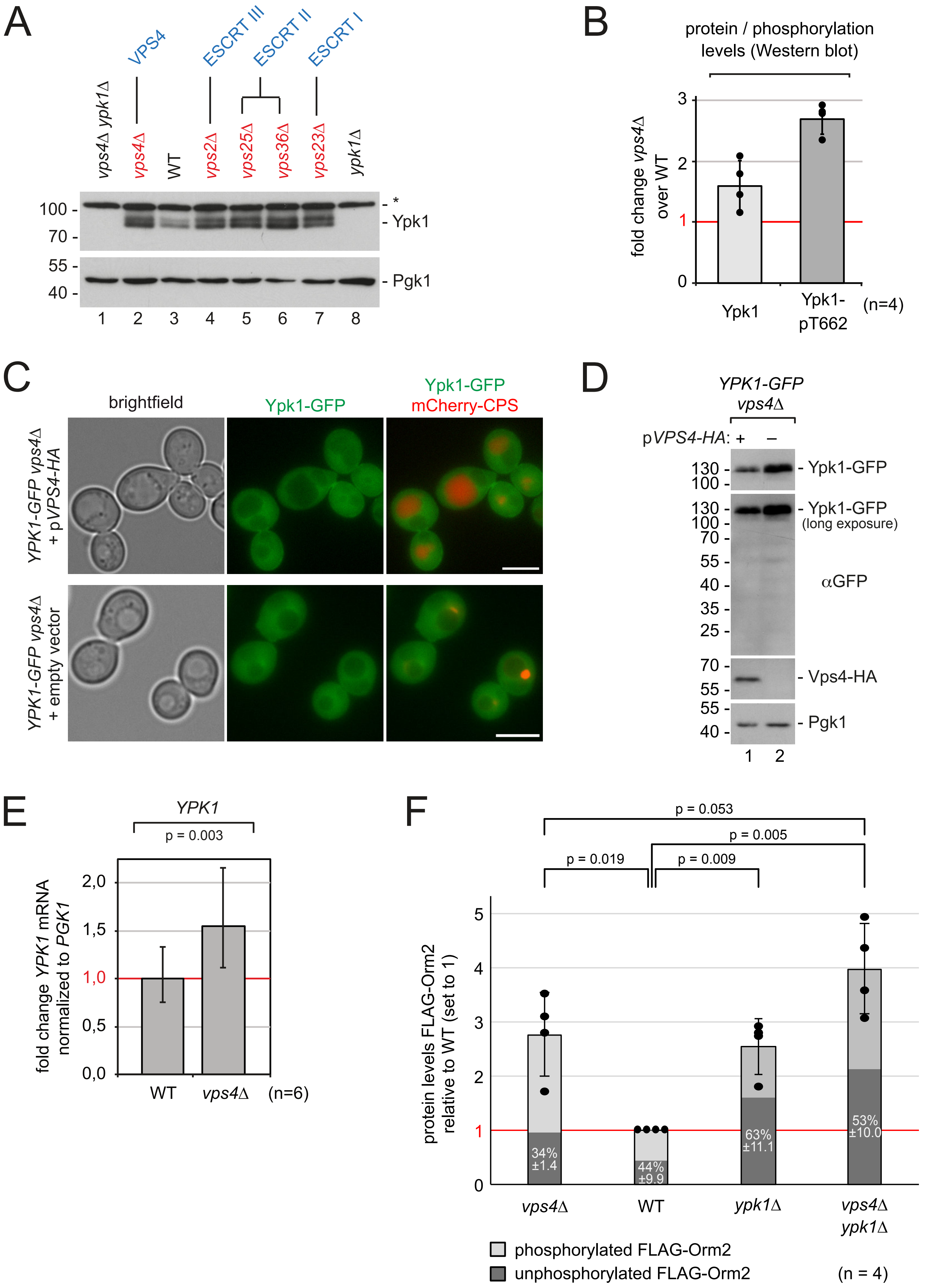

Figure S2 - related to Figure 3

Figure S2 - related to Figure 3.

(A) SDS-PAGE and Western blot analysis with the indicated antibodies of total yeast lysates from WT and the indicated mutants.

(B) Densitometric quantification of Ypk1 protein levels and Ypk1 pT662 phosphorylation levels in WT cells and *vps4*Δ mutants from Western blot experiments normalized to Pgk1 loading control. Data are presented as mean fold change in *vps4*Δ cells compared to WT levels ± standard deviation from 4 independent experiments. The circles indicate the individual measurements.

(C) Epifluorescence and phase contrast microscopy of living WT and *vps4*Δ cells expressing Ypk1-GFP (green) and the MVB cargo mCherry-CPS (red). Ypk1-GFP does not localize to the vacuole in WT or the class E compartment in ESCRT mutant cells grown under standard conditions. Scale bars 5μm.

(D) SDS-PAGE and Western blot analysis with the indicated antibodies of total yeast lysates from WT and *vps4*Δ cells expressing *YPK1-GFP*. Only full-length Ypk1-GFP protein was observed in WT cells and ESCRT mutants. The typical free GFP fragment at 25 kD, which would be indicative of vacuolar proteolysis, was not detected.

(E) Quantification of *YPK1* mRNA normalized to stable *PGK1* mRNA from WT cells and *vps4*Δ mutants by qPCR (n=6). Data are presented as mean fold change from WT ± standard deviation. Statistical significance was assessed by Student's t-test.

(F) Full bars: densitometric quantification of FLAG-Orm2 protein levels from Western blot analysis normalized to Pgk1 loading control from WT cells and the indicated mutants presented in Fig. 3B. Data are presented as mean fold change from WT ± standard deviation (n = 4). Statistical significance was assessed by Student's t-test. The circles indicate the individual measurements. The ratio between phosphorylated (light grey) and unphosphorylated FLAG-Orm2 (dark grey) was quantified densitometrically from Phos-tag Western blots. The percent values denote the fraction of unphosphorylated FLAG-Orm2 (mean ± standard deviation).

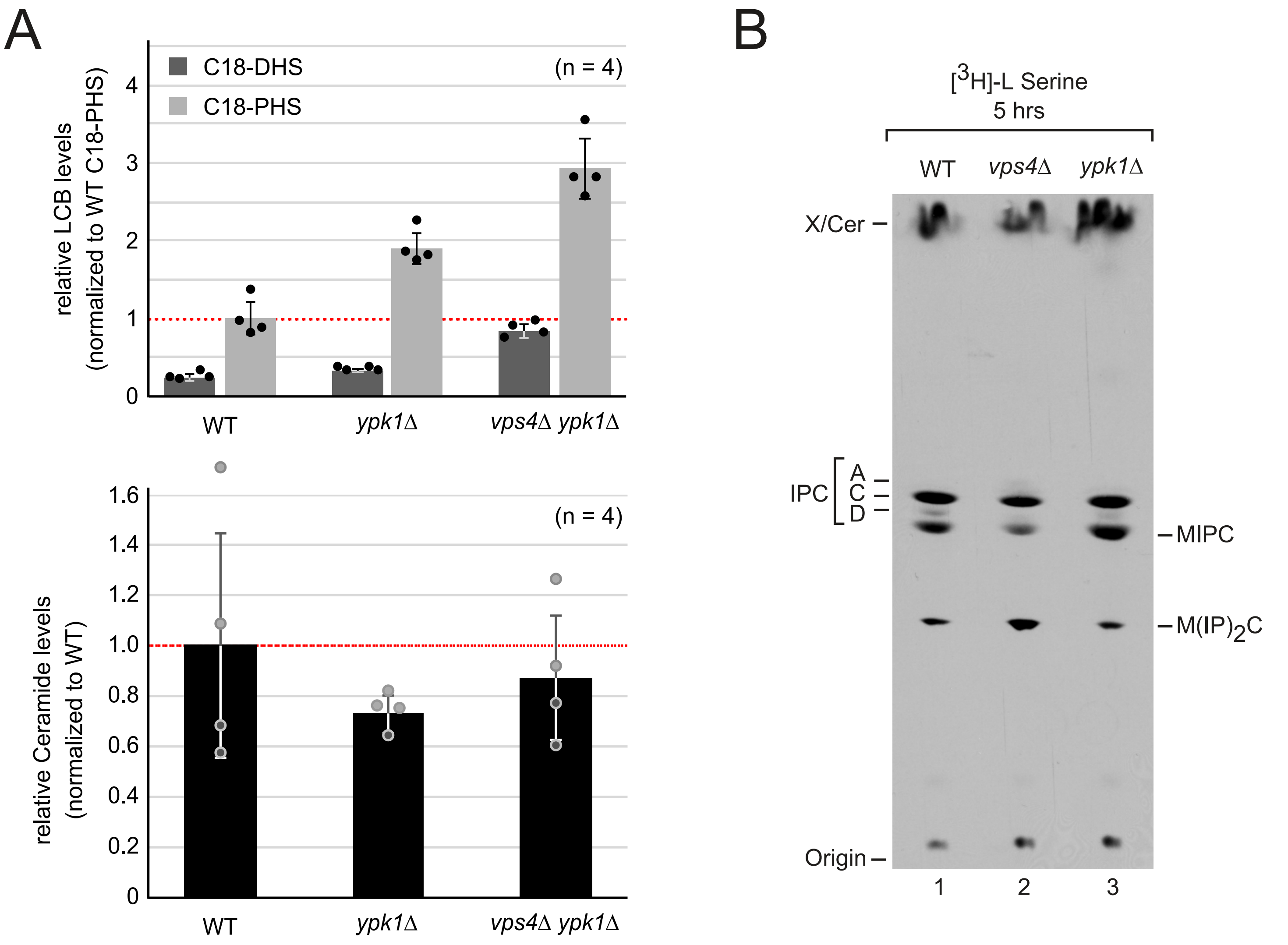

Figure S3 - related to Figure 3

Figure S3 - related to Figure 3.

(A) The levels of the long-chain bases (LCB) C18-dihydrosphingosine (C18-DHS) and C18-phytosphingosine (C18-PHS) and of ceramides (presented as the sum of all species) in lipid extracts from WT cells, *ypk1Δ* and *vps4*Δ *ypk1*Δ mutants were measured using LC-MS and quantified using non-yeast LCBs/ceramides as external standards. Data are normalized to WT levels of C18-PHS and ceramide (set to 1) and presented as mean ± standard deviation from 4 independent experiments. The circles indicate the individual measurements.

(B) Autoradiogram of sphingolipid extracts from [^{3}H]-serine radiolabeled WT cells and the indicated mutants that were separated by thin layer chromatography. X indicates an unknown lipid species that is insensitive to myriocin treatment (data not shown).

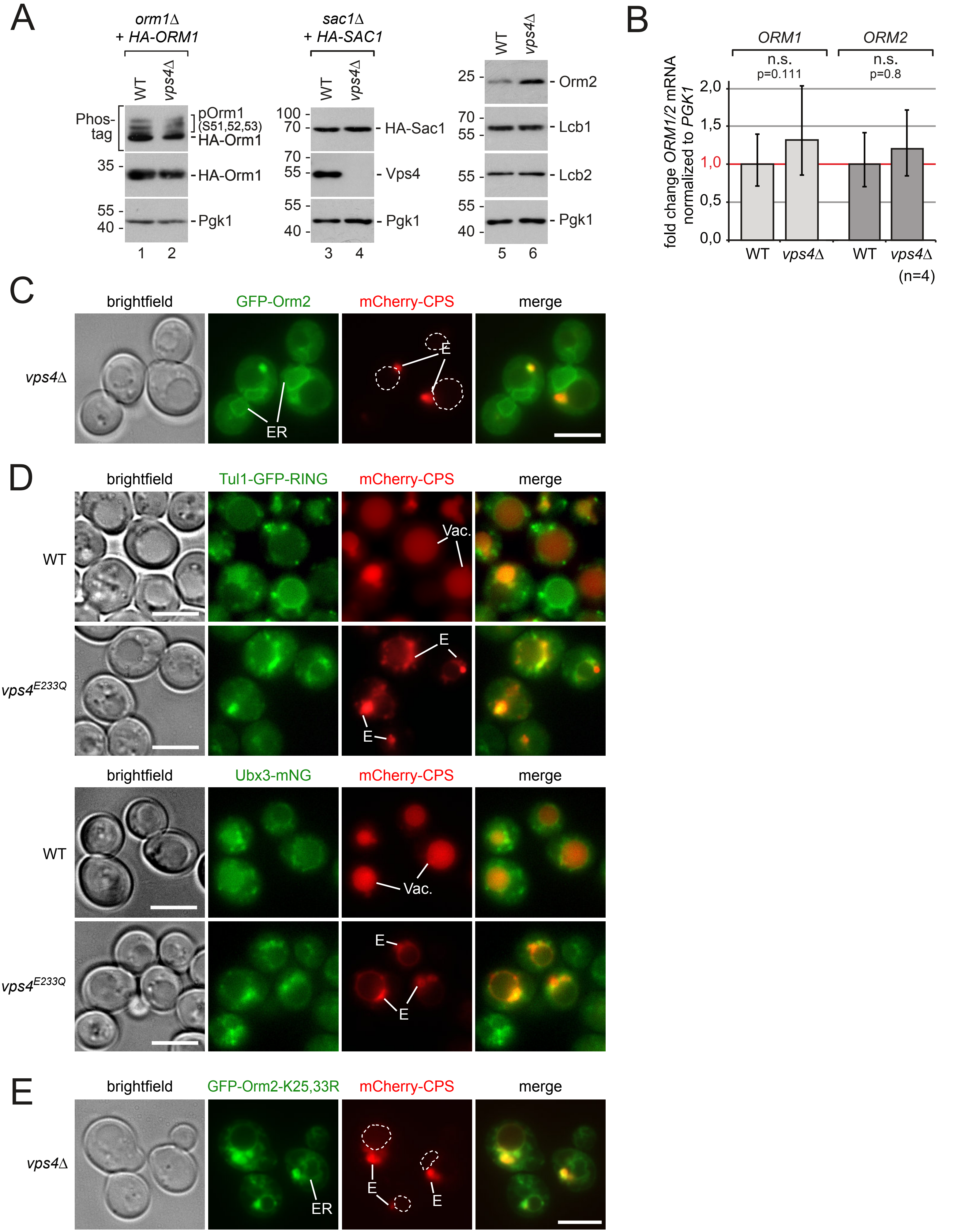

Figure S4 - related to Figure 4

Figure S4 - related to Figure 4.

(A) SDS-PAGE and Western blot analysis with the indicated antibodies of total yeast lysates from the indicated WT or *vps4*Δ strains.

(B) Quantification of *ORM1* and *ORM2* mRNA normalized to stable *PGK1* mRNA from WT cells and *vps4*Δ mutants by qPCR (n=4). Data are presented as mean fold change from WT ± standard deviation. Statistical significance was assessed by Student's t-test.

(C) Epifluorescence and phase contrast microscopy of living *vps4*Δ *(orm2*Δ*)* cells expressing GFP-Orm2 (green) and mCherry-CPS (red). ER, endoplasmic reticulum; E, class E compartment. Dashed circles indicate the position of vacuoles. Scale bars 5µm.

(D) Epifluorescence and phase contrast microscopy of living *tul1*Δ cells expressing *TUL1-GFP-RING* (green) or WT cells expressing Ubx3-mNeonGreen (green) and mCherry-CPS (red), and either dominant-negative *vps4*E233Q or empty plasmid as indicated. Vac, vacuole; E, class E compartment. Scale bars 5µm.

(E) Epifluorescence and phase contrast microscopy of living *vps4*Δ *(orm2*Δ*)* cells expressing GFP-Orm2-K25,33R (green) and mCherry-CPS (red). ER, endoplasmic reticulum; E, class E compartment. ER, endoplasmic reticulum; E, class E compartment. Dashed circles indicate the position of vacuoles. Scale bars 5µm.

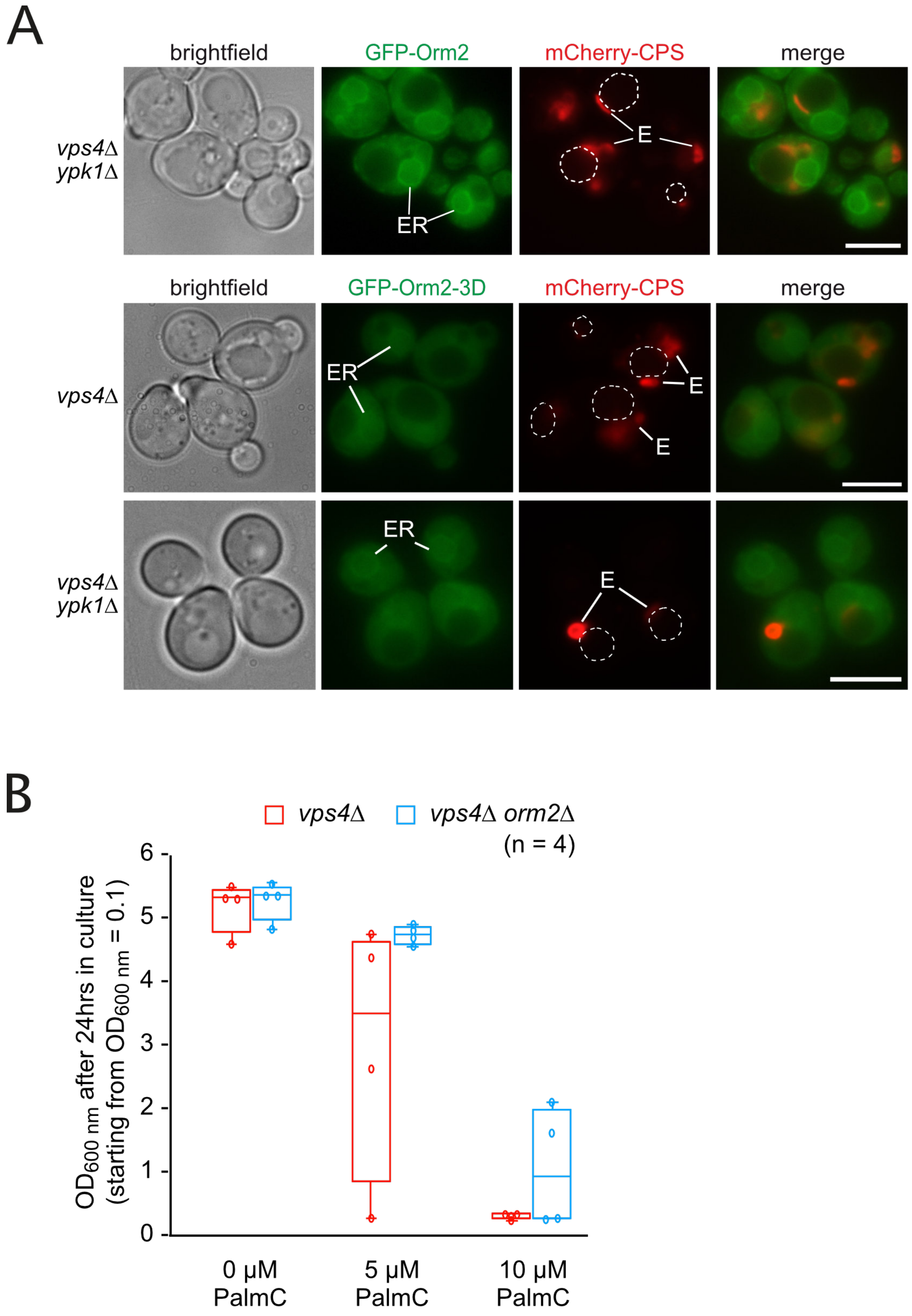

Figure S5 - related to Figure 5

Figure S5 - related to Figure 5.

(A) Epifluorescence and phase contrast microscopy of living *vps4*Δ and *vps4*Δ *ypk1*Δ cells (in *orm2*Δ background) expressing GFP-Orm2 or GFP-Orm2-3D (green) and mCherry-CPS (red). ER, endoplasmic reticulum; E, class E compartment. Dashed circles indicate the position of vacuoles. Scale bars 5µm.

(B) Growth of *vps4*Δ and *vps4*Δ *orm2*Δ cells in auxotrophic selection medium in presence of the indicated PalmC concentrations. Cells were inoculated to OD_{600nm} = 0.1 and grown for 24 hours in 4 independent experiments. The circles indicate the individual measurements.

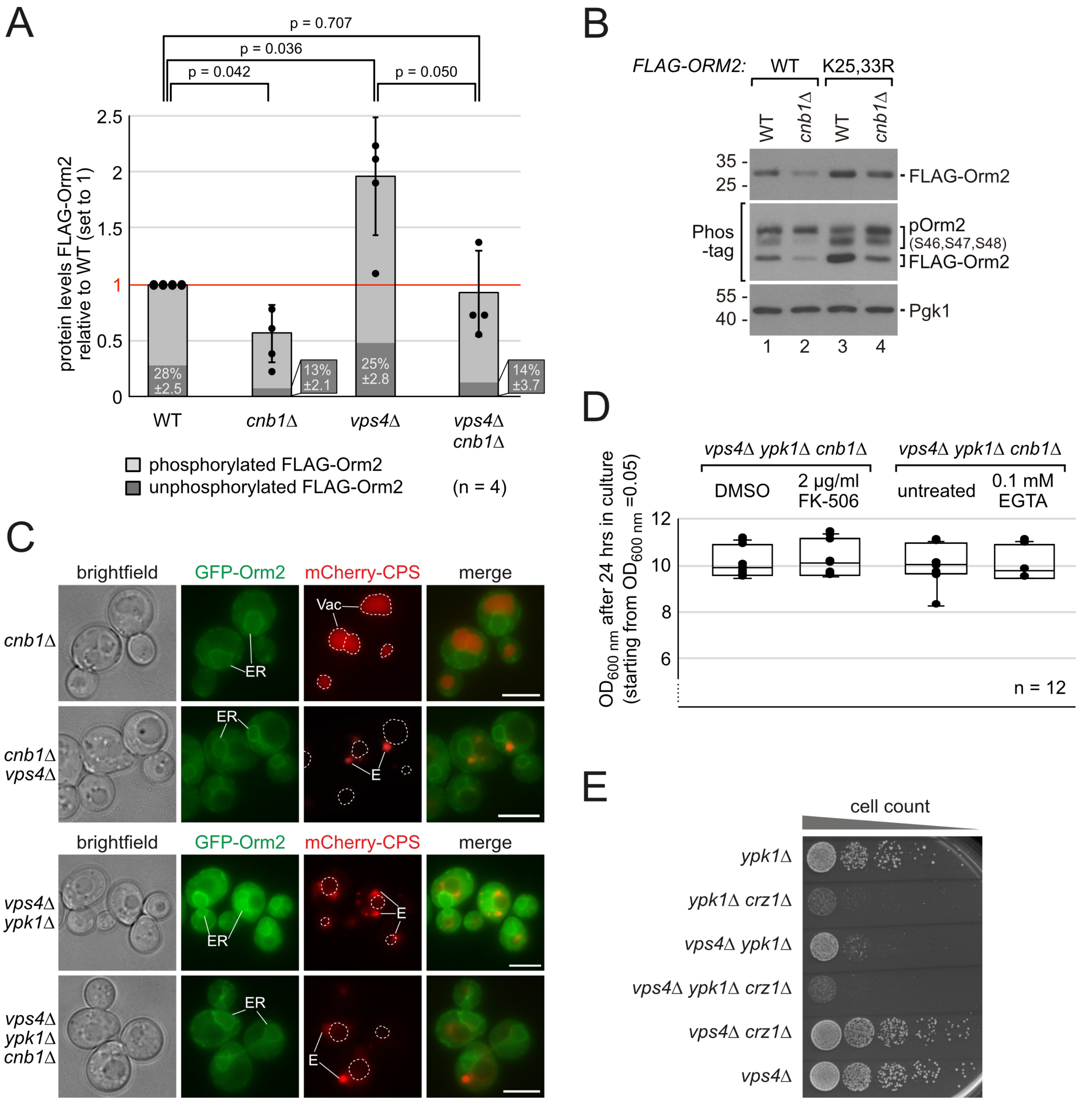

Figure S6 - related to Figure 6

Figure S6 - related to Figure 6.

(A) Full bars: densitometric quantification of FLAG-Orm2 protein levels from Western blot analysis normalized to Pgk1 loading control from WT cells and the indicated mutants presented in Fig. 6B. Data are presented as mean fold change from WT ± standard deviation (n = 4). Statistical significance was assessed by Student's t-test. The circles indicate the individual measurements. The ratio between phosphorylated (light grey) and unphosphorylated FLAG-Orm2 (dark grey) was quantified densitometrically from Phos-tag Western blots. The percent values denote the fraction of unphosphorylated FLAG-Orm2 (mean ± standard deviation).

(B) SDS-PAGE and Phos-tag SDS PAGE and Western blot analysis with the indicated antibodies of total yeast lysates from WT cells and *cnb1*Δ mutants expressing *FLAG- ORM2* or *FLAG-ORM2-K25,33R* (in *orm2*Δ background).

(C) Epifluorescence and phase contrast microscopy of living yeast cells from the indicated mutants (*cnb1*Δ and *vps4*Δ *cnb1*Δ are in an *orm2*Δ background) expressing GFP-Orm2 WT (green) and mCherry-CPS (red). ER, endoplasmic reticulum; E, class E compartment. Dashed circles indicate the position of vacuoles (Vac). Scale bars 5µm.

(D) Left: Growth of *vps4*Δ *ypk1*Δ *cnb1*Δ cells in YPD medium in presence of 2 µg/ml FK-506 or vehicle (DMSO). Right: Growth of *vps4*Δ *ypk1*Δ *cnb1*Δ cells in YPD medium in presence of 0.1 mM EGTA or untreated. Cells were inoculated to OD_{600nm} = 0.05 and grown for 24 hours in 3 independent experiments. The circles indicate the individual measurements (12 technical replicates).

(E) Equal amounts of the indicated single, double or triple mutants in serial dilutions were incubated on a YPD agar plate at 26°C.

Table S1: Gene ontology term 'molecular processes' enrichment analysis

GOID	GO term	Frequency	Genome Frequency	adj. p-value	enrichment	Gene(s)
**GO:0042592**	**homeostatic process**	**15 out of 119 genes, 12.6%**	**276 of 6433 genes, 4.3%**	**4,85E-03**	**2,94**	**CSG2,VMA2,PER1,VMA1,NHX1,VMA3,PMR1,VMA7, VMA10,ICE2,VPS53,VMA5,RAD50,PHO80,VMA4**
**GO:0006629**	**lipid metabolic process**	**15 out of 119 genes, 12.6%**	**296 of 6433 genes, 4.6%**	**5,06E-03**	**2,74**	**DEP1,CSG2,PER1,UME6,CAX4,HTD2,OAR1,YPK1,SAC1, ERG6,ERG5,PAH1,ERG2,ERG24,MCT1**
**GO:0006351**	**transcription, DNA-templated**	**23 out of 119 genes, 19.3%**	**673 of 6433 genes, 10.5%**	**2,01E-02**	**1,85**	**DEP1,CCR4,RRN10,NHP6B,RXT2,SRB8,HPR1,UME6,HTA1 ,RAD4,SNF6,PFD1,NNT1,SFP1,GIM5,CTK3,MKS1,SIN4, YNL296W,PHO80,THP1,CKA2,SSN3**
GO:0006464	cellular protein modification process	20 out of 119 genes, 16.8%	665 of 6433 genes, 10.3%	7,35E-02	1,63	DEP1,GCV3,PER1,UBX3,NBP2,GDA1,CAX4,TUL1,ELM1, YPK1,LIP2,NNT1,STE11,CTK3,YDJ1,URE2,PHO80,CKA2, SSN3,PPT2
GO:0040007	growth	8 out of 119 genes, 6.7%	165 of 6433 genes, 2.6%	7,35E-02	2,62	RXT2,SEC66,UME6,PDA1,ELM1,STE11,MKS1,SSN3
GO:0006950	response to stress	20 out of 119 genes, 16.8%	672 of 6433 genes, 10.4%	7,35E-02	1,61	DEP1,HTA2,SHP1,TPS1,HPR1,SAC3,ACL4,NBP2,HTA1, EAF1,RAD4,SNF6,YLR235C,IMH1,MMS22,STE11,YDJ1, RAD50,THP1,CKA2
GO:0051276	chromosome organization	17 out of 119 genes, 14.3%	554 of 6433 genes, 8.6%	7,84E-02	1,66	DEP1,TPD3,HTA2,SHP1,NHP6B,RXT2,UBX3,SAC3,UME6, HTA1,EAF1,RAD4,SNF6,NNT1,MMS22,RAD50,THP1
GO:0016192	vesicle-mediated transport	12 out of 119 genes, 10.1%	351 of 6433 genes, 5.5%	8,51E-02	1,85	SLA1,CDC50,UBX3,SWA2,VMA3,GLO3,PMR1,EMP24, VPS53,IMH1,END3,SLA2
GO:0006461	protein complex assembly	10 out of 119 genes, 8.4%	282 of 6433 genes, 4.4%	8,82E-02	1,92	SLA1,PIM1,VMA2,EAF1,VMA21,ICE2,ELM1,GIM5,PKR1, MDM12
GO:0006412	translation	7 out of 119 genes, 5.9%	727 of 6433 genes, 11.3%	8,82E-02	0,52	TPD3,RPS9B,DHH1,RPS24A,RPL34B,MRPL15,CTK3
GO:0005975	carbohydrate metabolic process	7 out of 119 genes, 5.9%	198 of 6433 genes, 3.1%	1,17E-01	1,91	DEP1,SHP1,TPS1,UME6,SNF6,ELM1,EXG1
GO:0061024	membrane organization	9 out of 119 genes, 7.6%	282 of 6433 genes, 4.4%	1,17E-01	1,73	SEC66,CDC50,GLO3,ICE2,YPK1,IMH1,PAH1,PHO80, MDM12
GO:0006259	DNA metabolic process	13 out of 119 genes, 10.9%	453 of 6433 genes, 7.0%	1,17E-01	1,55	DEP1,CCR4,HTA2,HPR1,SAC3,HTA1,EAF1,RAD4,HUR1, SNF6,MMS22,RAD50,THP1
GO:0007010	cytoskeleton organization	8 out of 119 genes, 6.7%	238 of 6433 genes, 3.7%	1,17E-01	1,82	SLA1,SAC6,ICE2,PFD1,ELM1,END3,SRV2,SLA2
GO:0034655	nucleobase-containing compound catabolic process	5 out of 119 genes, 4.2%	146 of 6433 genes, 2.3%	1,39E-01	1,85	CCR4,DHH1,SAC3,THP1,SSN3
GO:0007049	cell cycle	14 out of 119 genes, 11.8%	644 of 6433 genes, 10.0%	1,39E-01	1,18	TPD3,CCR4,SHP1,BUD31,SAC3,UME6,ELM1,MMS22, END3,SLA2,RAD50,YNL296W,PHO80,THP1
GO:0044281	small molecule metabolic process	15 out of 119 genes, 12.6%	675 of 6433 genes, 10.5%	1,39E-01	1,20	DEP1,GCV3,UME6,RNR1,PDA1,HTD2,CYC1,ADO1,ELM1, OAR1,ERG6,ERG5,ERG2,ERG24,MCT1
GO:0071554	cell wall organization or biogenesis	6 out of 119 genes, 5.0%	198 of 6433 genes, 3.1%	1,39E-01	1,64	NBP2,PRS3,EXG1,STE11,END3,SLA2
GO:0006091	generation of precursor metabolites and energy	5 out of 119 genes, 4.2%	154 of 6433 genes, 2.4%	1,39E-01	1,76	SHP1,CYC1,OAR1,YLR294C,PAH1
GO:0007005	mitochondrion organization	2 out of 119 genes, 1.7%	265 of 6433 genes, 4.1%	1,39E-01	0,41	YDJ1,MDM12
GO:0051301	cell division	5 out of 119 genes, 4.2%	127 of 6433 genes, 2%	1,39E-01	2,13	BUD31,ELM1,END3,SLA2,THP1
GO:0000003	reproduction	12 out of 119 genes, 10.1%	467 of 6433 genes, 7.3%	1,39E-01	1,39	SHP1,RXT2,UME6,ELM1,YPK1,MMS22,STE11,END3,SLA2, RAD50,YNL296W,CKA2
GO:0042254	ribosome biogenesis	5 out of 119 genes, 4.2%	416 of 6433 genes, 6.5%	1,45E-01	0,65	RPS9B,SAC3,RPS24A,SNF6,DBP7
GO:0006913	nucleocytoplasmic transport	5 out of 119 genes, 4.2%	180 of 6433 genes, 2.8%	1,72E-01	1,50	HPR1,SAC3,YDJ1,URE2,THP1
GO:0006397	mRNA processing	4 out of 119 genes, 3.4%	170 of 6433 genes, 2.6%	2,09E-01	1,27	BUD31,HPR1,SAC3,THP1
GO:0030154	cell differentiation	4 out of 119 genes, 3.4%	160 of 6433 genes, 2.5%	2,09E-01	1,35	SHP1,END3,YNL296W,CKA2
GO:0007165	signal transduction	5 out of 119 genes, 4.2%	240 of 6433 genes, 3.7%	2,09E-01	1,13	NBP2,STE11,MKS1,SRV2,PHO80
GO:0055085	transmembrane transport	3 out of 119 genes, 2.5%	234 of 6433 genes, 3.6%	2,09E-01	0,69	VMA2,SEC66,NHX1
GO:0048856	anatomical structure development	4 out of 119 genes, 3.4%	160 of 6433 genes, 2.5%	2,09E-01	1,35	SHP1,ELM1,END3,YNL296W
GO:0006605	protein targeting	5 out of 119 genes, 4.2%	307 of 6433 genes, 4.8%	2,09E-01	0,88	SEC66,VMA3,ICE2,YDJ1,URE2
GO:0007059	chromosome segregation	3 out of 119 genes, 2.5%	203 of 6433 genes, 3.2%	2,34E-01	0,80	TPD3,SHP1,MMS22
GO:0007034	vacuolar transport	2 out of 119 genes, 1.7%	168 of 6433 genes, 2.6%	2,34E-01	0,64	VMA3,VPS53
GO:0048646	anatomical structure formation involved in morphogenesis	3 out of 119 genes, 2.5%	137 of 6433 genes, 2.1%	2,34E-01	1,18	SHP1,END3,YNL296W
GO:0006790	sulfur compound metabolic process	2 out of 119 genes, 1.7%	122 of 6433 genes, 1.9%	2,71E-01	0,89	PDA1,MET10
GO:0006457	protein folding	2 out of 119 genes, 1.7%	95 of 6433 genes, 1.5%	2,71E-01	1,14	PFD1,YDJ1

Table S2: Yeast strains, plasmids and reagents

Antibodies	SOURCE	IDENTIFIER
Goat anti mouse IgG-Peroxidase	Sigma	Cat. # A4416; RRID:AB_258167
Goat anti rabbit IgG-Peroxidase	Sigma	Cat. # A0545; RRID:AB_257896
Rabbit anti goat IgG-Peroxidase	Sigma	Cat. # A5420; RRID:AB_258242
Mouse monoclonal anti PGK1 (22C5D8)	Invitrogen	Cat. # 459250; RRID:AB_2532235
Mouse monoclonal anti GFP (IgG1K, clones 7.1 and 13.1)	Roche Diagnostics	Cat. # 11814460001; RRID:AB_390913
Goat anti-GFP polyclonal	Rockland	Cat. # 600-101-215; RRID:AB_218182
Rabbit anti goat Fab' 1.4 nm NANOGOLD™	Nanoprobes	Cat. # 2004
Mouse monoclonal anti FLAG M2	Sigma	Cat. # F3165; RRID:AB_259529
Mouse monoclonal anti HA (12CA5)	Abcam	Homemade hybridoma supernatant, lot 20081201
Mouse monoclonal anti ubiquitin (P4D1)	Santa Cruz	Cat. # sc-8017; RRID:AB_628423
Mouse monoclonal anti Ypk1 phospho-T662	Gift from Robbie Loewith	(Berchtold et al., 2012)
Goat polyclonal anti-Ypk1	Santa Cruz	Cat. # yl15; RRID:AB_793265
Rabbit polyclonal anti-Orm2, generated against N-terminal peptide MIDRTKNESPAFEESPLTP	Gift from Howard Riezman	This study
Rabbit polyclonal anti-Lcb1	Gift from Teresa Dunn	(Gable, Slife et al., 2000)
Rabbit polyclonal anti-Lcb2	Gift from Teresa Dunn	(Gable et al., 2000)
Rabbit polyclonal anti-Tul1	Gift from Peter Espenshade	(Tong et al., 2014)
Rabbit polyclonal anti-Ubx3	Gift from Peter Espenshade	(Tong et al., 2014)
Rabbit polyclonal anti-Dsc2	Gift from Peter Espenshade	(Tong et al., 2014)
Rabbit polyclonal anti-Dsc3	Gift from Peter Espenshade	(Tong et al., 2014)
Rabbit polyclonal anti-Vps4	Gift from Scott Emr	(Babst et al., 1998)

Chemicals, Peptides, and Recombinant Proteins	SOURCE	IDENTIFIER
[3H]-L-serine	Hartmann analytics	Cat. # MT910
anti-Flag magnetic beads M2	Sigma	Cat. # M8823; RRID:AB_2637089
3xFLAG peptide	Sigma	Cat. # F4799
Phos-tag acrylamide	Wako	Cat. # 304-93521
Cycloheximide	Sigma	Cat. # C7698
Myriocin	Sigma	Cat. # M1177-5MG
Rapamycin	LC Laboratories	Cat. # R-5000
Palmitoyl carnitine	Sigma	Cat. # 91503-10MG
Propidium iodide	Sigma	Cat. # 81845

Table S2: Yeast strains, plasmids and reagents (continued)

FK-506	Sigma	Cat. # E3889
Ethylene glycol-bis(2-aminoethylether)-N,N,N,N′ -tetraacetic acid (EGTA)	Sigma	Cat. # 3779
**Commercial Assays**	**SOURCE**	**IDENTIFIER**
TAQman gene expression assay for ORM1	Thermo Scientific	Cat. # Sc04125000_s1
TAQman gene expression assay for ORM2	Thermo Scientific	Cat. # Sc04149509_s1
TAQman gene expression assay for YPK1	Thermo Scientific	Cat. # Sc04141261_s1
TAQman gene expression assay for PGK1	Thermo Scientific	Cat. # Sc04104844_s1
RNeasy Mini kit	Qiagen	Cat. # 74104
Revert Aid First strand cDNA synthesis kit	Thermo	Cat. # K1622
**Yeast strains:**	**SOURCE**	**IDENTIFIER**
SEY6210 wildtype (*MATalpha leu2-3, 112 ura3-52 his3-200 trp1-901 lys2-801 suc2-9)*	(Robinson, Klionsky et al., 1988)	SEY6210
SEY6210.1 wildtype (*MATa leu2-3, 112 ura3-52 his3-200 trp1-901 lys2-801 suc2-9)*	(Robinson et al., 1988)	SEY6210.1
SEY6210 *vps4::TRP1*	(Babst et al., 1997)	MBY3
SEY6210.1 *vps4::TRP1*	(Babst et al., 1997)	MBY4
SEY6210.1 *vps23::HIS3*	(Babst et al., 2000)	MBY23
SEY6210.1 *vps36::HIS3*	(Babst et al., 2000)	MBY13
SEY6210.1 *vps2::HIS3*	this study, (Babst et al., 2002a)	DTY521
SEY6210.1 *vps25::HIS3*	this study, (Babst et al., 2002b)	OSY396
SEY6210.1 *tul1::HIS3*	(Schmidt et al., 2019)	SSY17
SEY6210.1 *orm1::HIS3*	(Schmidt et al., 2019)	OSY510
SEY6210 *orm2::TRP1*	(Schmidt et al., 2019)	YSC580
SEY6210.1 *orm2::TRP1*	(Schmidt et al., 2019)	OSY774
SEY6210.1 *orm2::TRP1 tul1::HIS3*	(Schmidt et al., 2019)	YWY027
SEY6210.1 *vps4::TRP1 orm2::TRP1*	(Schmidt et al., 2019)	VBY51
SEY6210.1 *vps4::TRP1 tul1::HIS3 orm2::TRP1*	(Schmidt et al., 2019)	YWY029
SEY6210.1 *tul1::HIS3 orm2::TRP1 YIP(TRP1)-dsRed-HDEL*	(Schmidt et al., 2019)	OSY725
SEY6210.1 *orm2::TRP1 ypk1::HIS3*	this study	VBY61
SEY6210.1 *orm2::TRP1 ypk1::HIS3 vps4::TRP1*	this study	VBY60
SEY6210.1 *orm1::HIS3 vps4::TRP1*	this study	OSY520
SEY6210.1 *orm1::HIS3 ypk1::HIS3 vps4::TRP1*	this study	OSY492
SEY6210.1 *ypk1::HIS3*	this study	MMY059
SEY6210.1 *ypk1::HIS3 vps4::TRP1*	this study	OSY323
SEY6210.1 *sac1::HIS3*	this study	OSY566
SEY6210.1 *sac1::HIS3 vps4::TRP1*	this study	OSY570
SEY6210.1 *csg2::HIS3*	this study	OSY529
SEY6210.1 *csg2::HIS3 vps4::TRP1*	this study	OSY531
SEY6210.1 *erg2::HIS3*	this study	OSY568
SEY6210.1 *erg2::HIS3 vps4::TRP1*	this study	OSY572

Table S2: Yeast strains, plasmids and reagents (continued)

SEY6210.1 *ypk2::HIS3*	this study	OSY397
SEY6210.1 *ypk2::HIS3 vps25::HIS3*	this study	OSY398
SEY6210.1 *ypk1::HIS3 vps25::HIS3*	this study	OSY393
SEY6210.1 *ypk2::HIS3 vps2::HIS3*	this study	OSY401
SEY6210.1 *ypk1::HIS3 vps2::HIS3*	this study	OSY394
SEY6210 *ypk1::HIS3*	this study	OSY402
SEY6210.1 *chm7::TRP1*	this study	OSY577
SEY6210.1 *chm7::TRP1 ypk1::HIS3*	this study	OSY579
SEY6210.1 *YPK1-GFP::HIS3 vps4::TRP1*	this study	MMY075
TB50xSEY *vps4::TRP1 tor1-1 avo3Δ1274-1430::hphMX6, Mat.A*	this study, (Gaubitz et al., 2015)	OSY486
TB50xSEY *vps4::TRP1 tor1-1, Mat.A*	this study, (Gaubitz et al., 2015)	OSY490
SEY6210.1 *orm2::TRP1 vps25::HIS3*	this study	YWY016
SEY6210 *UBX3-mNeonGreen::TRP1*	this study, (Yang et al., 2018)	MWY218
SEY6210.1 *cnb1::HIS3*	this study	OSY274
SEY6210.1 *vps4::TRP1 cnb1::HIS3*	this study	OSY291
SEY6210 *cnb1::HIS3 ypk1::HIS3*	this study	OSY426
SEY6210 *vps4::TRP1 cnb1::HIS3 ypk1::HIS3*	this study	OSY428
SEY6210.1 *orm2::TRP1 cnb1::HIS3*	this study	OSY908
SEY6210.1 *orm2::TRP1 vps4::TRP1 cnb1::HIS3*	this study	OSY910
SEY6210.1 *vps4::TRP1 crz1::HIS3*	this study	OSY466
SEY6210 *crz1::HIS3 ypk1::HIS3*	this study	OSY499
SEY6210.1 *vps4::TRP1 crz1::HIS3 ypk1::HIS3*	this study	OSY497

**Primer for gene knock out / knock in**	**Used for strain:**	**Primer name:**
GTTCCTCTCATATCAACAAACATTAATACAGTTCCTGAAAC GGATCCCCGGGTTAATTAA	OSY323	YPK1_KO_F
CATCATTCCTGAACTTGAGGTGGCATCATTGGGTGTCCCTG AATTCGAGCTCGTTTAAAC	OSY323	YPK1_KO_R
TGGGTCAAATTATCGCGTATACAAATATACATATAGTAACC GGATCCCCGGGTTAATTAA	OSY397	YPK2_KO_F
TAAAATAGTAGAGTTAGAGGTGATAGGGAAGGGAAGAGTAG AATTCGAGCTCGTTTAAAC	OSY397	YPK2_KO_R
CACCACCAAAGCCAGCCAAATAGTATCTCACTGTATCTGAC GGATCCCCGGGTTAATTAA	OSY577	CHM7_KO_F
CCCACTTTAACACTAGCAATTCGAAGGTCATTTTCTGTGAT GCGAATTCGAGCTCGTTTAAAC	OSY577	CHM7_KO_R2
TCGCTCAATCAAACTAAGACTAGCCCAGACCATTATAGCCC GGATCCCCGGGTTAATTAA	OSY568	ERG2_KO_F
GGACTACCGCATGACTGATTTCGTGAGGTCGGGCAGCTTAG AATTCGAGCTCGTTTAAAC	OSY568	ERG2_KO_R
GCTGGTGAGTTAGCACGATAACAAACAAAGATACAGCGTCC GGATCCCCGGGTTAATTAA	OSY529	CSG2-KO-F
CAAGAATGGGTCAGAAAGTTCACCCGTGTTAGGGTTGACGG AATTCGAGCTCGTTTAAAC	OSY529	CSG2-KO-R
TTTAGTCTCGATTGGAAGTTTCGTCAGACAGTACAAGGAAG CGGATCCCCGGGTTAATTAA	OSY466	CRZ1_KO_F
AAAAAAAAATTCCTATTCAAAGCTTAAAAAAACAAAAATAA GAATTCGAGCTCGTTTAAAC	OSY466	CRZ1_KO_R
CAGTAACGATAATATTTATATACACGTATATTTTCTCGTCT AGATCGGATCCCCGGGTTAATTAA	OSY566	SAC1_KO_F2
CAGCCCAGTATATTGGCACAGATCCTCTTGTCTGTAAGAAG GAGGAATTCGAGCTCGTTTAAAC	OSY566	SAC1_KO_R2

Table S2: Yeast strains, plasmids and reagents (continued)

ATGGTGATCAGAATCCATAGAAGCATTTTTATTTCTTAAACGGATCCCCGGGTTAATTAA	OSY274	CNB1_KO_F
AAATCCACGTCCCCACTATTATCAGCATCGAAAACCTCCAGAATTCGAGCTCGTTTAAAC	OSY274	CNB1_KO_R
**Primer for plasmid cloning**	**Used for plasmid(s):**	**Primer name:**
gcgGGATCCACTTCCTGAACCTTTGTATAGTTCATCC	pOS218	GFP-Orm2-BamHI-R
cgcaacgttGCTGACGTCTTCTTGAAACCCGTC	pVB01	YPK1_HindIII_F
gcgctcgagGGATCTCGGAAGGGTAAGAGAGG	pVB01	YPK1_XhoI_R
GGTTTCAATAAGATATCTACCTTTGCTGTGACAAGGAAATTACCTTATTTAAAG	pOS096, pOS097	Ypk1_D242A_F
CTTTAAATAAGGTAATTTCCTTGTCACAGCAAAGGTAGATATCTTATTGAAACC	pOS096, pOS097	Ypk1_D242A_R
GATCgtcgacGCTGAAGAATACTTGAAGCC	pVB03	SalI_YPK2_fw
CATGactagtCTTCGCTTTGACATCCGC	pVB03	SpeI_YPK2_rev
gcgTCTAGAGTTTATGTATGTGTTTTTTGTAGTTATAGATTTAAGC	pOS246	Prom-TPI1_XbaI_R
cgcGAGCTCGTACTGTCATCATCGTTTGC	pYW036	TUL1-SacI-F
gcgACTAGTGGAGTTGGAGCGATTTAG	pYW036	TUL1-SpeI-R
CGAGTATATATCGGAGCATGGTAGTAAAGGAGAAGAACTTTTCACTGG	pYW036	TUL1-pH-RING-F1
CCAGTGAAAAGTTCTTCTCCTTTACTACCATGCTCCGATATATACTCG	pYW036	TUL1-pH-RING-R1
GGCATGGATGAACTATACAAAGGTGGAACTGCTGAACATACC	pYW036	TUL1-pH-RING-F2
GGTATGTTCAGCAGTTCCACCTTTGTATAGTTCATCCATGCC	pYW036	TUL1-pH-RING-R2
**Plasmids**	**SOURCE**	**IDENTIFIER**
empty centromer vector *HIS3*	(Sikorski & Hieter, 1989)	pRS413
empty centromer vector *TRP1*	(Sikorski & Hieter, 1989)	pRS414
empty centromer vector *LEU2*	(Sikorski & Hieter, 1989)	pRS415
empty centromer vector *URA3*	(Sikorski & Hieter, 1989)	pRS416
pRS415 - *VPS4* (endogenous 5' and 3')	(Müller et al., 2015)	pOS014
pRS416 - *VPS4* (endogenous 5' and 3')	(Müller et al., 2015)	pMM22
pRS415 - *VPS4-3xHA* (endogenous 5' and 3')	(Adell et al., 2014)	pOS063
pRS413 - *VPS4-3xHA* (endogenous 5' and 3')	(Adell et al., 2014)	pDT74
pRS416 - *3xFLAG-ORM2* (endogenous 5' and 3')	(Schmidt et al., 2019)	pOS129
pRS415 - *HA-SAC1* (endogenous 5' and 3')	(Schmidt et al., 2019)	pOS166
pRS415 - *GFP-ORM2* (endogenous 5' and 3')	(Schmidt et al., 2019)	pOS173
pRS416 - *GFP-ORM2* (endogenous 5' and 3') (BamHI site)	this study	pOS218
pRS416 - *ORM2* (endogenous 5' and 3')	(Schmidt et al., 2019)	pVB17
pRS416 - *3xFLAG-ORM2*^{S46A,S47A,S48A} (endogenous 5' and 3')	(Schmidt et al., 2019)	pYW001
pRS416 - *3xFLAG-ORM2*^{S46D,S47D,S48D} (endogenous 5' and 3')	(Schmidt et al., 2019)	pYW002
pRS416 - *3xFLAG-ORM2*^{K25,33R} (endogenous 5' and 3')	(Schmidt et al., 2019)	pYW015
pRS416 - *GFP-ORM2* (endogenous 5' and 3')	(Schmidt et al., 2019)	pYW006
pRS416 - *GFP-ORM2*^{K25R,K33R} (endogenous 5' and 3')	(Schmidt et al., 2019)	pYW028

Table S2: Yeast strains, plasmids and reagents (continued)

pRS416 - *GFP-ORM2*^{S46A,S47A,S48A} (endogenous 5' and 3')	(Schmidt et al., 2019)	pYW008
pRS416 - *GFP-ORM2*^{S46D,S47D,S48D} (endogenous 5' and 3')	(Schmidt et al., 2019)	pYW009
pRS415 - *VPS4-3xHA-eGFP* (endogenous 3' and 5')	(Adell et al., 2017)	pSS37
pRS415 - *P*^{TDH3}*-mCherry-CPS1(2-270)*	(Adell et al., 2014)	pC29
pRS416 - *P*^{TDH3}*-mCherry-CPS1*	(Adell et al., 2014)	pC6
pRS414 - *P*^{TDH3}*-mCherry-CPS1(2-270)*	this study	pMM29
pRS415 - *P*^{TPI1}*-mCherry-CPS1*	this study	pOS246
pRS416 - *YPK1* (endogenous 5' and 3')	this study	pVB01
pRS416 - *YPK2* (endogenous 5' and 3')	this study	pVB03
pRS415 - *YPK1-13xmyc* (endogenous 5' and 3')	this study, Courtesy from C. Stefan	pOS109
pRS415 - *YPK1-13xmyc T504A* (endogenous 5' and 3')	this study, Courtesy from C. Stefan	pOS110
pRS415 - *YPK1-13xmyc D242A* (endogenous 5' and 3')	this study	pOS096
pRS415 - *YPK1-13xmyc S644A T662A* (endogenous 5' and 3')	this study, Courtesy from C. Stefan	pOS111
pRS415 - *YPK1-13xmyc S644A T662A D242A* (endogenous 5' and 3')	this study	pOS097
pRS415 - *P*^{ADH1}*-GFP-SAC1*	this study, Courtesy from C. Stefan	pOS229
pRS415 - *SNF7-LAP-eGFP* (endogenous 5' and 3')	(Adell et al., 2017)	pSM35
pRS416 - *3xHA-ORM1* (endogenous 5' and 3')	(Schmidt et al., 2019)	pOS127
pRS415 - *TUL1-GFP-RING* (endogenous 5' and 3')	this study	pYW036
pRS415 - *VPS4*-E233Q	(Adell et al., 2014)	pOS015
pRS416 - *VPS4*-E233Q	(Müller et al., 2015)	pDT84
**Software and Algorithms**	**SOURCE**	**IDENTIFIER**
Photoshop CS5	Adobe	Version 12.0.4x64; RRID:SCR_014199
Illustrator CS5.1	Adobe	Version 15.1.0; RRID:SCR_010279
Affinity Photo	Serif	Version 1.7.3 RRID:SCR_016951
Affinity Designer	Serif	Version 1.7.3 RRID:SCR_016952
Excel for Mac	Microsoft	Version 16.16.2; RRID:SCR_016137
ImageJ2	(Rueden et al., 2017)	Version 2.0.0-rc49/1.51h; RRID:SCR_003070
Fiji	(Schinderlin et al., 2012)	Version 1.0
Visi View	Visitron	Version 2.1.4
Attune^{TM} NxT software	Life Technologies	v. 3.1.1243.0
